# Supplementary material for: Noncollinear Electric Dipoles in a Polar Chiral Phase of CsSnBr3 Perovskite
Source: J Am Chem Soc. 2024 May 31;146(23):15701–17. doi: 10.1021/jacs.4c00679 (PMC11177262; doi:10.1021/jacs.4c00679)
Supplement: Supplementary file 1 — ja4c00679_si_001.pdf [file ja4c00679_si_001.pdf]

Supporting Information
Noncollinear electric dipoles in a polar, chiral phase of $CsSnBr_3$ perovskite

Douglas H. Fabini,*[a,b] Kedar Honasoge,[a] Adi Cohen,[c] Sebastian Bette,[a] Kyle M. McCall,[d,e] Constantinos C. Stoumpos,[f] Steffen Klenner,[g] Mirjam Zipkat,[h] Le Phuong Hoang,[a] Jürgen Nuss,[a] Reinhard K. Kremer,[a] Mercouri G. Kanatzidis,[d] Omer Yaffe,[c] Stefan Kaiser,[a] Bettina V. Lotsch[a,h]

[a] Max Planck Institute for Solid State Research, 70569 Stuttgart, Germany
[b] Department of Chemistry, Massachusetts Institute of Technology, Cambridge, Massachusetts 02139, United States
[c] Department of Chemical and Biological Physics, Weizmann Institute of Science, Rehovot 76100, Israel
[d] Department of Chemistry, Northwestern University, Evanston, Illinois 60208, United States
[e] Present Address: Department of Materials Science and Engineering, University of Texas at Dallas, Richardson, Texas 75080, United States
[f] Department of Materials Science and Technology, University of Crete, Vassilika Voutes, 70013 Heraklion, Greece
[g] Institut für Anorganische und Analytische Chemie, Universität Münster, 48149 Münster, Germany
[h] Department of Chemistry, Ludwig-Maximilians-Universität, 81377 München, Germany
* E-mail: fabini@mit.edu

Contents

1 Laboratory powder X-ray diffraction (PXRD) 2

2 High temperature heat capacity detail 3

3 ^{119}Sn Mössbauer spectroscopy 4

4 First-principles phonon mode mapping 5

5 Comparison of the proposed structures of phases I and II with reported perovskite structures 9

6 Symmetry relations between the crystal structures of phases I−V 10

7 Chemical trends of acentric Sn(II) coordination 12

8 ^{81}Br nuclear quadrupole resonance (NQR) spectroscopy 14

9 Synchrotron powder diffraction 15

10 Polarization−orientation-dependence of Raman scattering from birefringent crystals in point group 2 23

11 Raman spectra and analysis 27

12 Comparison of experimental and calculated structures 30

13 Strain impacts on lone pair distortions 31

14 Temperature evolution of lattice parameters 32

15 Electronic structure impacts of distortions 34

16 ^{119}Sn nuclear magnetic resonance (NMR) spectroscopy 35

17 Electronic conductivity comparison between $CsGeBr_3$, $CsSnBr_3$, and $CsPbBr_3$ 37

1 Laboratory powder X-ray diffraction (PXRD)

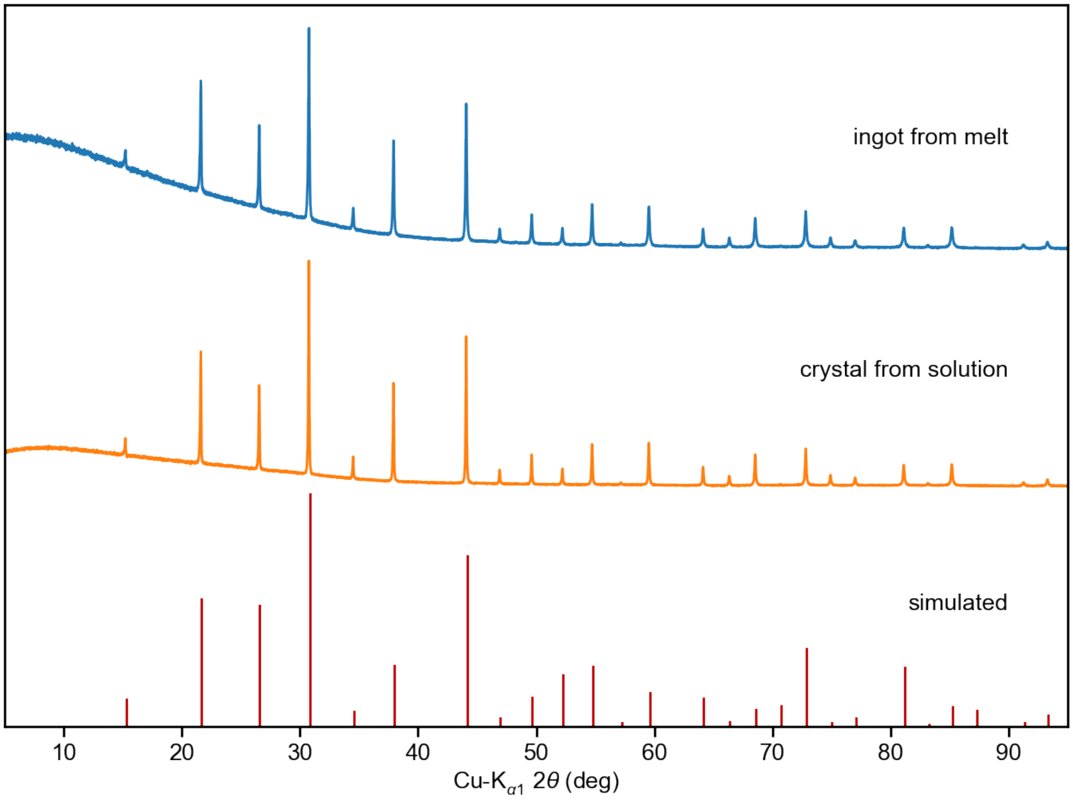

Figure S1: Powder X-ray diffraction (Cu-K$_{\alpha1}$) for crushed $CsSnBr_3$ ingot solidified from the melt and $CsSnBr_3$ crystals grown from ethylene glycol solution. Red ticks indicate the Bragg reflection angles and intensities expected for the reported room temperature, cubic phase.[1]

2 High temperature heat capacity detail

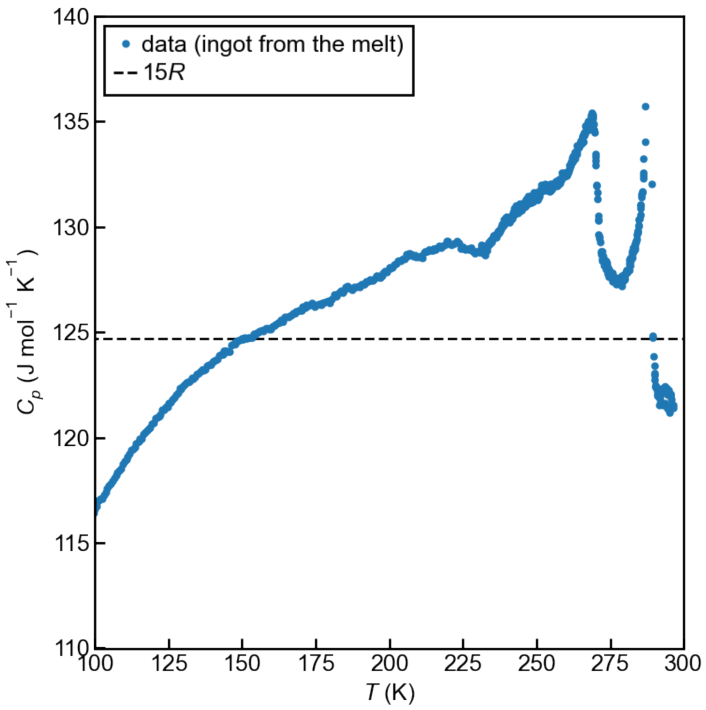

Figure S2: Detailed view of the specific heat of polycrystalline $CsSnBr_3$ from the melt. While there are no clear phase transitions between 100 K and 269 K (the temperature of the phase III−phase IV transition), there is some structure to the data around 230 K. The heat capacity exceeds the classical limit of Dulong and Petit above 150 K in phase III reflecting significantly anharmonic structural dynamics.

3 ^{119}Sn Mössbauer spectroscopy

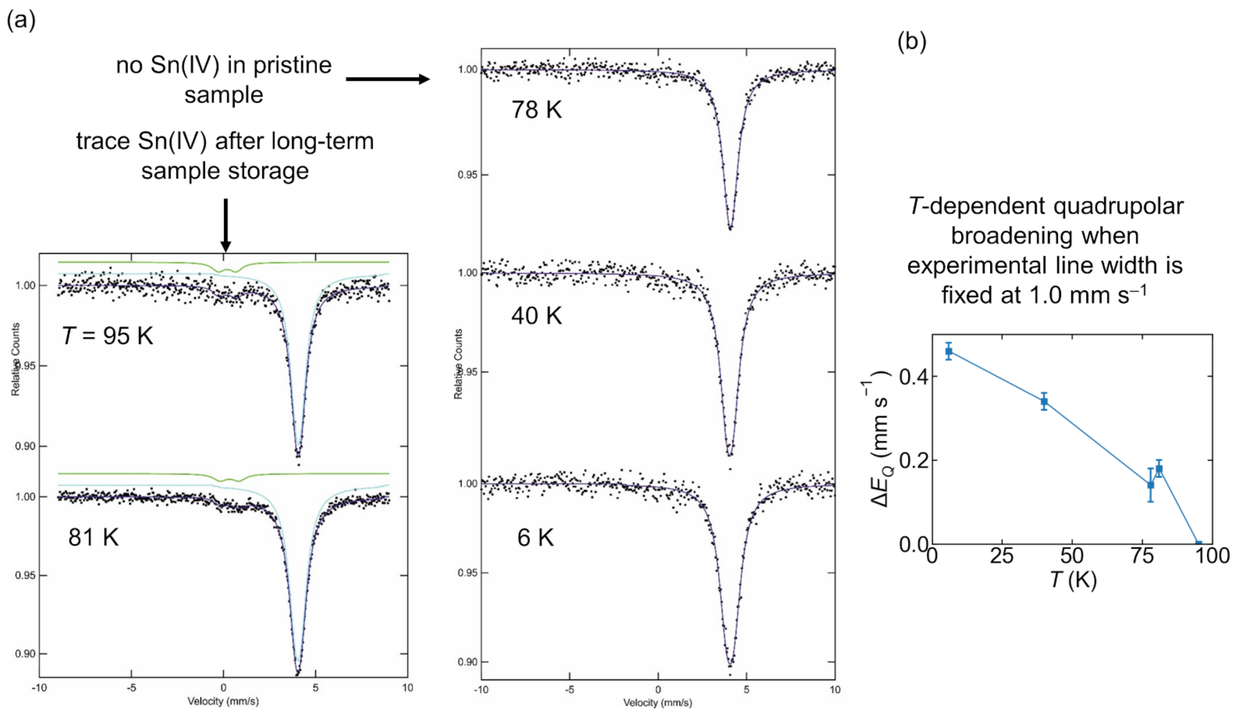

Figure S3: (a) ^{119}Sn Mössbauer spectra of $CsSnBr_3$ at several temperatures. Trace Sn(IV) is visible at the highest temperatures, which were recorded at a later date. In all spectra, the Sn(II) signal broadens slightly on cooling, consistent with unresolved site splitting, quadrupolar splitting, or both in phases I and II. (b) This subtle broadening can be seen clearly by fixing the line width and allowing the quadrupolar broadening to vary. Fitting parameters are given in Table S1.

Table S1: Fitting parameters of ^{119}Sn Mössbauer spectroscopic measurements for $CsSnBr_3$. δ = isomer shift, ΔE_Q = quadrupole splitting, Γ = experimental line width. (*) The line width for the majority Sn(II) signal was fixed to 1.0 mm s^{-1} because the splitting is insufficiently large to resolve the difference between line width, multiple sites, and quadrupolar splitting. A trace Sn(IV) environment is detectable at the highest temperatures, which were measured at a later date. These Sn(IV) contributions indicate partial oxidation of the sample.

T (K)	δ (mm s^{-1})	ΔE_Q (mm s^{-1})	Γ (mm s^{-1})
95	4.060(6)	0	1.0*
	0.20(8)	0.98(13)	0.8(2)
81	4.038(3)	0.18(2)	1.0*
	0.33(5)	1.03(8)	0.86(13)
78	4.086(5)	0.14(4)	1.0*
40	4.045(6)	0.34(2)	1.0*
6	4.064(6)	0.46(2)	1.0*

4 First-principles phonon mode mapping

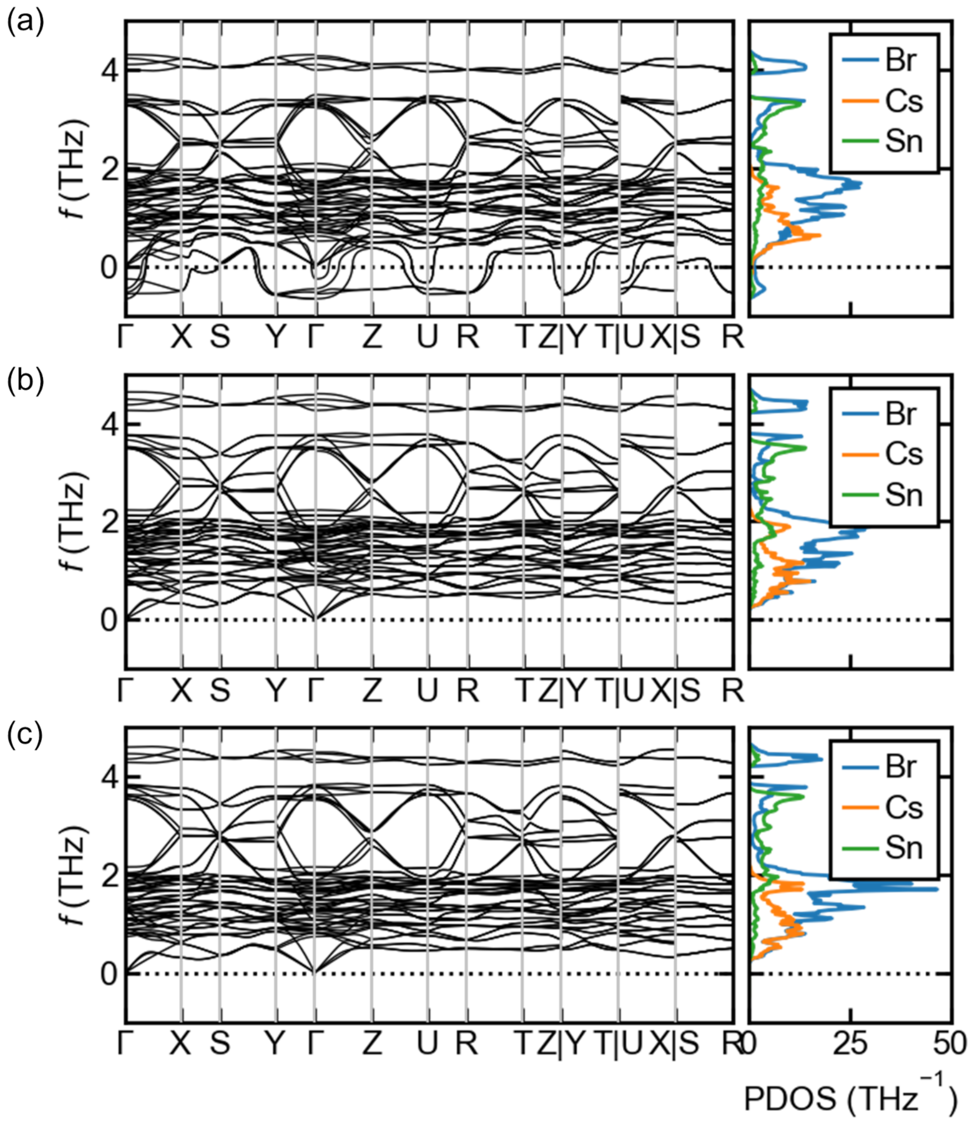

Figure S4: Sensitivity of calculated phonons to the exchange−correlation functional and dispersion corrections. Harmonic phonon dispersions and atom-projected densities of states (PDOS) for the orthorhombic phase III of $CsSnBr_3$ with (a) PBE, (b) PBE+vdW (DFT-D3), and (c) PBEsol functionals. All are computed with a $\sqrt{2} \times 1 \times \sqrt{2}$ supercell ($2 \times 2 \times 2$ with respect to the cubic phase V). Only PBE without van der Waals corrections correctly finds instabilities.

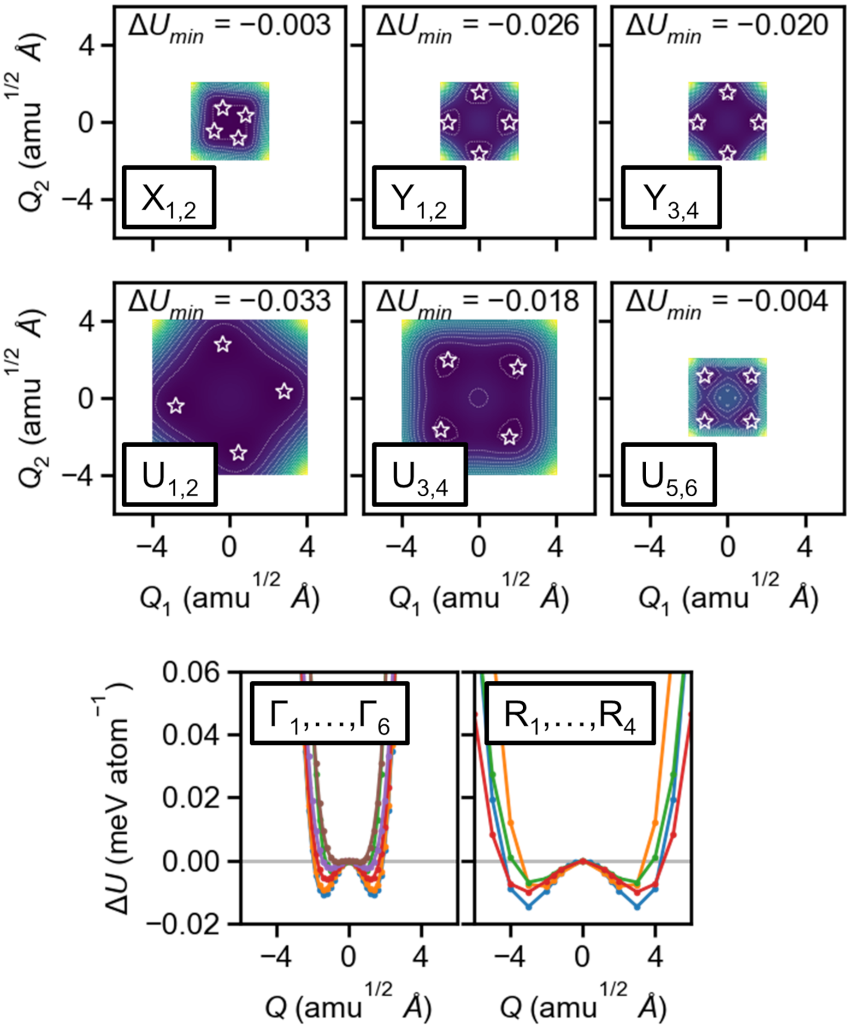

Figure S5: Potential energy surfaces computed along unstable phonon eigenvectors for phase III of $CsSnBr_3$ (space group $Pnma$, #62). The first six panels show heatmaps of the total energy for linear combinations of doubly-degenerate unstable phonon modes at high symmetry points, with indices counting up from the mode with the most negative frequency. The doubly-degenerate imaginary modes at Z (not pictured) do not to lead to new minima and their apparent instability may be an artifact.[2] The wavevectors, positions of the new minima (white stars), and the minimum energies (ΔU_{min}, in meV atom^{-1}) before relaxation are indicated. The lower two panels are for six singly-degenerate modes at Γ and four degenerate modes at R (which were only explored in one dimension as a simplifying assumption as discussed in the text).

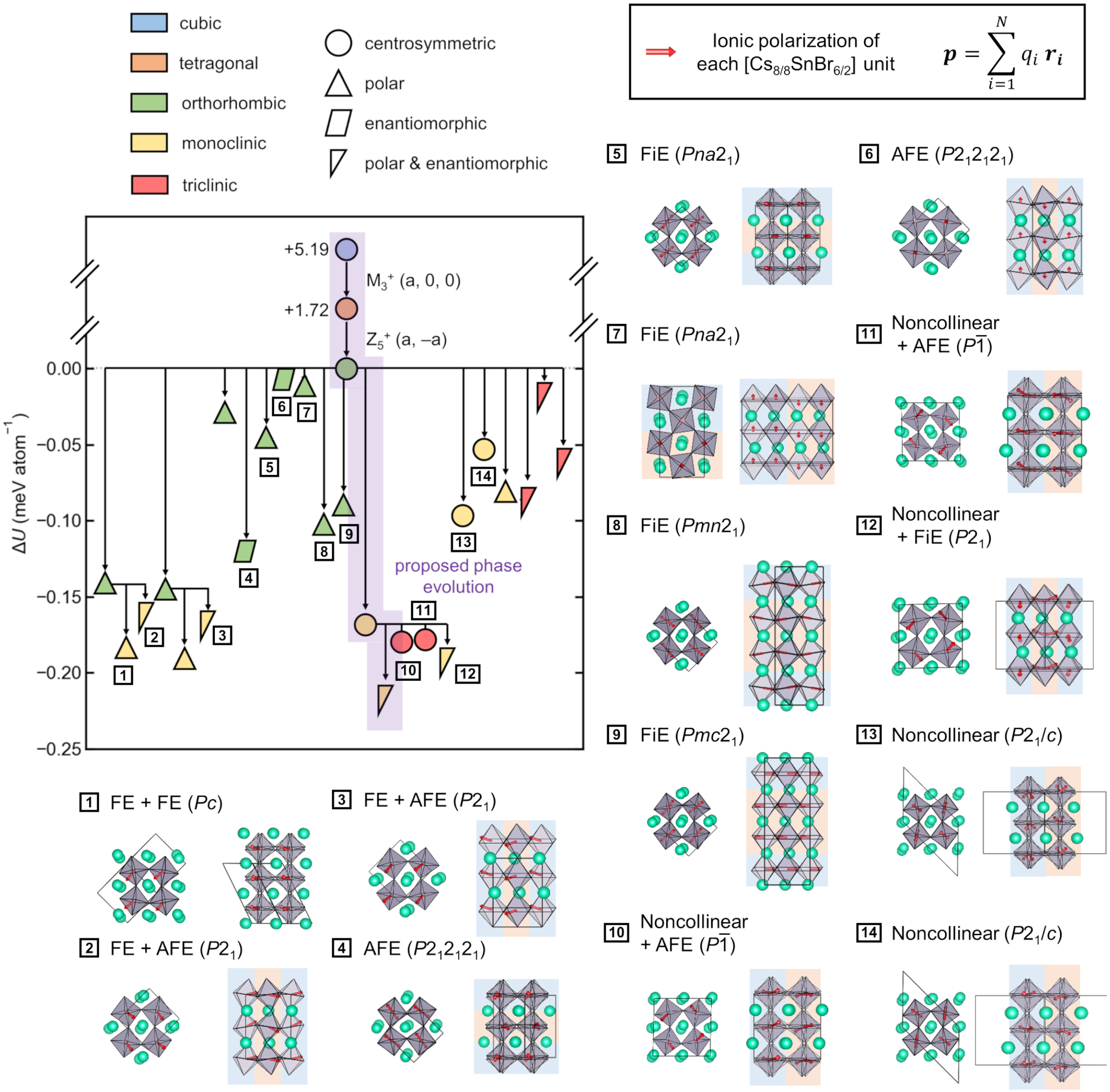

Figure S6: Visualization of additional candidate structures generated by first-principles phonon mode-mapping, beyond those depicted in the main text. The ordering of local dipoles is described as well as the resulting space group symmetry ("FE" = ferroelectric, "AFE" = antiferroelectric, "FiE" = ferrielectric). Red arrows indicate cluster-resolved local electric dipoles computed in the ionic limit. For some candidates, blue and orange backgrounds aid the eye in seeing stripes of different local polarization directions.

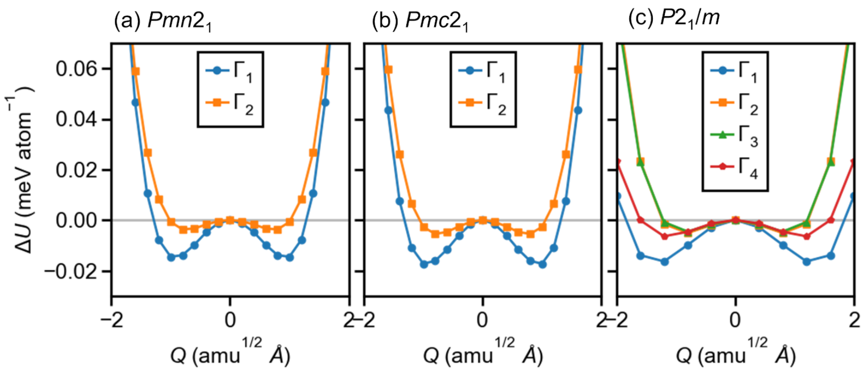

Figure S7: Potential energy surfaces computed along unstable Γ-point phonon eigenvectors for the three lowest energy candidates for $CsSnBr_3$ phase II: (a) $Pmn2_1$, from relaxation of the new saddle point along the phonon labeled Γ_1 in Figure S5; (b) $Pmc2_1$, from relaxation of the new saddle point along the phonon labeled Γ_2 in Figure S5; and (c) $P2_1/m$, from relaxation of the new saddle point along the linear combination of degenerate phonons labeled $U_{1,2}$ in Figure S5. The Γ_1 phonon eigenvectors are similar in the three cases (ferroic distortions along the in-phase tilt axis) and lead to similar reductions in energy (both here and after relaxation of all primary and secondary order parameters), but the $P2_1/m$ phase is rather lower in energy to begin with (see Figure S6 and main text).

5 Comparison of the proposed structures of phases I and II with reported perovskite structures

Table S2: Comparison of the proposed structures of $CsSnBr_3$ phases I and II with tabulated structures (ICSD) with perovskite connectivity, matching space group and Pearson symbol, and identical or similar Wyckoff sequences. For phase I, two isopointal entries are found, but these phases are not isoconfigurational[3] to $CsSnBr_3$, as evident in their different superstructures. For phase II, no isopointal entries are found. The structures of $CsSnBr_3$ phases I and II are thus new types.

Space group	Pearson symbol	Wyckoff sequence	Composition	Notes
$P2_1$	$mP40$	a^{20}	$CsSnBr_3$, $T < 77$ K	**Proposed phase I**
$P2_1$	$mP40$	a^{20}	$BiMnO_3$ (local structure model from neutron pair distribution function)	Isopointal, but not isoconfigurational: Different superstructure
$P2_1$	$mP40$	a^{20}	$YBaMn_2O_6$	Isopointal, but not isoconfigurational: Different superstructure
$P2_1/m$	$mP40$	f^6e^8	$CsSnBr_3$, 77 K $< T <$ 85 K	**Proposed phase II**
$P2_1/m$	$mP40$	f^4e^8dcba	La_2CuSnO_6	Different coloring variant of common supergroup, leading to different superstructure
$P2_1/m$	$mP20$	f^2e^4da	$CsSnBr_3$, $T = 85$ K	**Proposed phase III−phase II intermediate**
$P2_1/m$	$mP20$	f^2e^4da	$(Nd,Ca)_xMnO_3$	Isopointal and isoconfigurational

6 Symmetry relations between the crystal structures of phases I–V

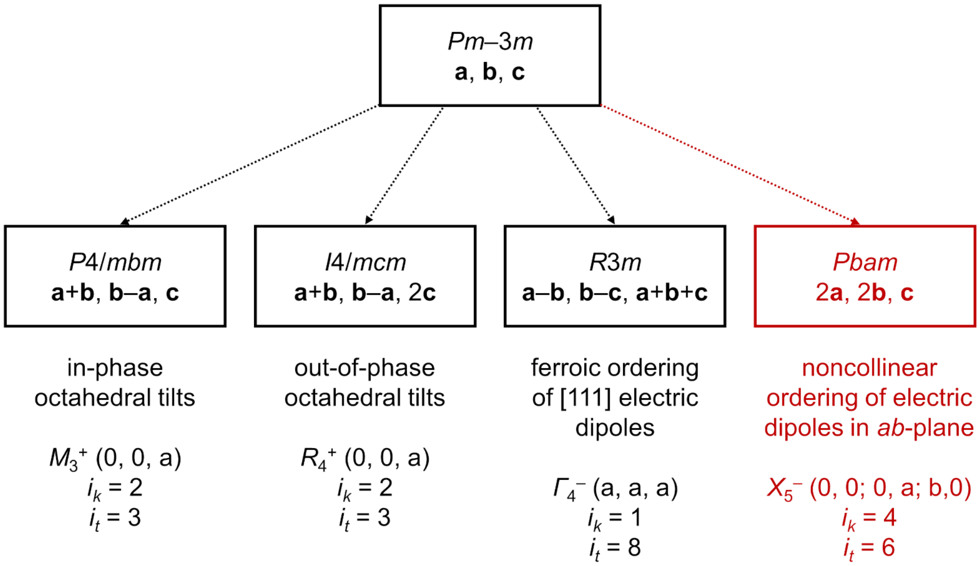

Figure S8: Example distortions from the cubic perovskite aristotype. Distortions for each of the three irreps considered by Stokes and coworkers[4] along an arbitrary order parameter direction are given in black, including the change in unit cell, space group symmetry, and the index of the subgroup (i_k = *klassengleich* index, i_t = *translationengleich* index). The same information is presented in red for the new type of noncollinear distortion we find in $CsSnBr_3$. Combinations of the three irreps considered by Stokes and coworkers lead to 61 unique structures.[4] Future group theoretic analysis could additionally consider the X_5^- irrep, presumably leading to many hundreds or thousands of possible structures.

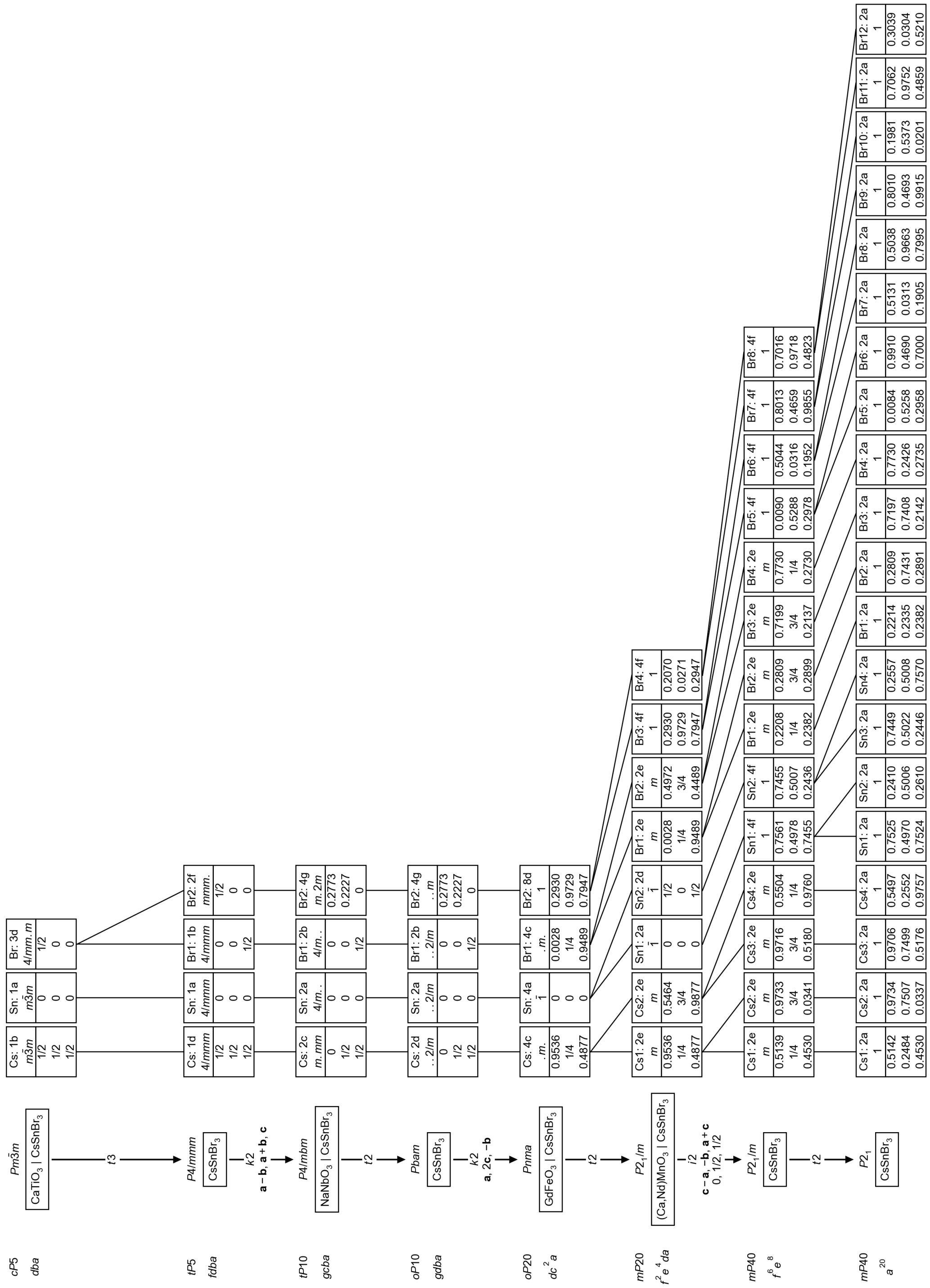

Figure S9: Group-subgroup scheme in the Bärnighausen formalism[5–8] for the different polymorphs of $CsSnBr_3$. The indices for the *translationengleiche* (t), *isomorphic* (i), and *klassengleiche* (k) symmetry reductions and the evolution of the atomic parameters are given. The column at the left lists the Pearson codes and the Wyckoff sequences for each superstructure variant. The present tree is an extension of the group-subgroup scheme originally presented by Bock and Müller.[9]

7 Chemical trends of acentric Sn(II) coordination

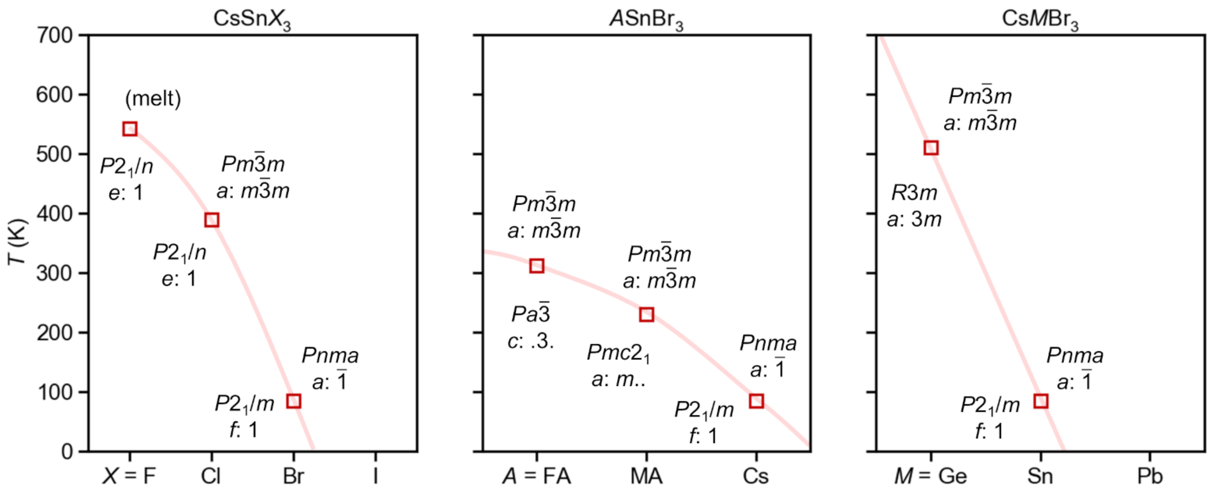

Figure S10: Transition temperatures between the highest temperature solid phase with acentric Sn(II) coordination and the phase above. Data are shown with respect to chemical substitution on all three sites: halide, A-site cation, and octahedral group 14 cation. For each composition, the space group symmetry and the Sn Wyckoff position and site symmetry are annotated for the phases above and below the transition to acentric Sn(II) coordination. FA = formamidinium, MA = methylammonium. Lines are to guide the eye only. Note that several of these phases with acentric Sn(II) environments nonetheless exhibit inversion symmetry, and some break inversion symmetry on cooling further (e.g. $CsSnBr_3$). Reported structures and transition temperatures are taken for $CsSnF_3$,[10] $CsSnCl_3$,[11], $FASnBr_3$,[12], $MASnBr_3$,[13], and $CsGeBr_3$.[14]

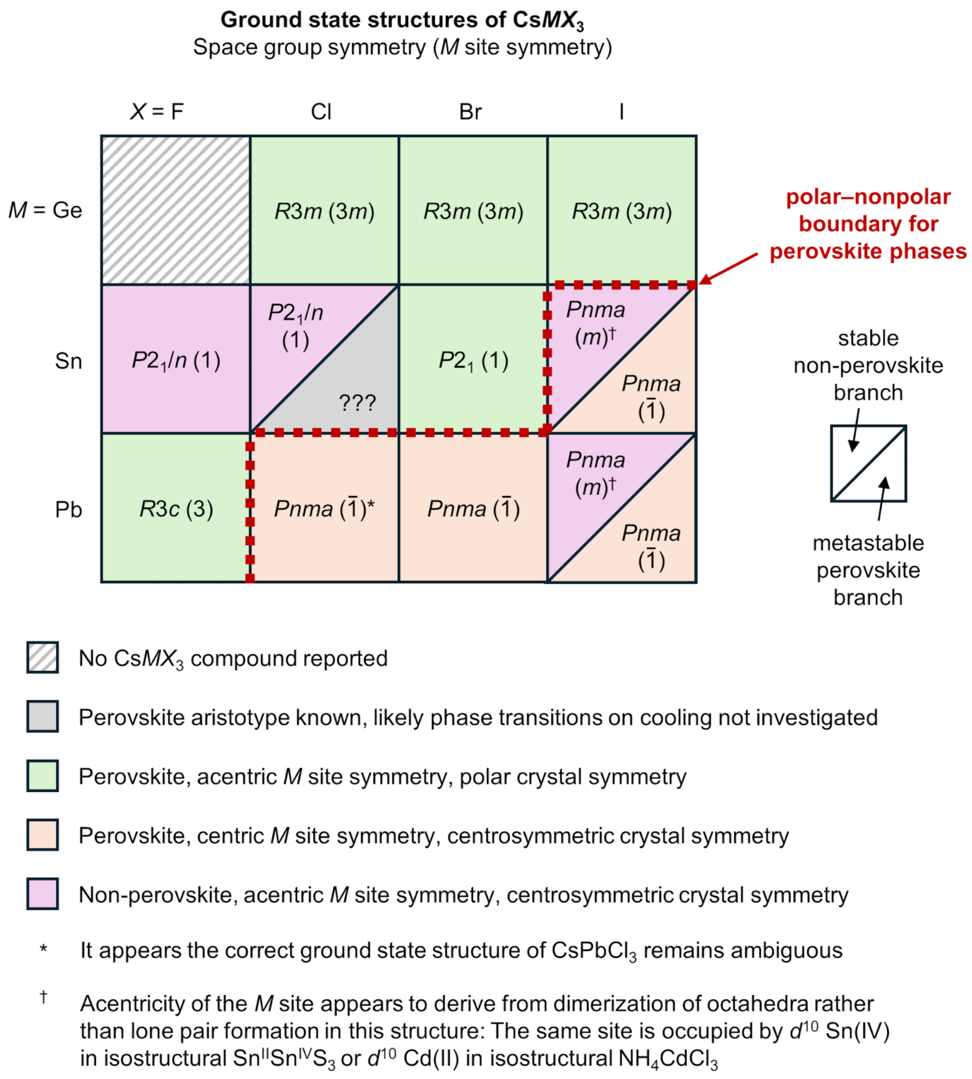

Figure S11: Reported ground state crystal structures and octahedral cation site symmetries for $CsMX_3$ compounds. Prior to this work, the boundary between polar and nonpolar perovskite phases appeared to lie between Ge^{2+} and Sn^{2+} for all halides except F^-. In light of our findings for $CsSnBr_3$, it is likely that the metastable perovskite phase of $CsSnCl_3$ exhibits one or more phase transitions on cooling to a structure which combines tilting and lone pair distortions. Structures are taken from the literature for $CsGeX_3$,[14] $CsSnF_3$,[10] $CsSnCl_3$,[11] $CsSnI_3$,[15,16] $CsPbF_3$,[17] $CsPbCl_3$,[18–20] $CsPbBr_3$,[21] and $CsPbI_3$.[22,23]

8 ^{81}Br nuclear quadrupole resonance (NQR) spectroscopy

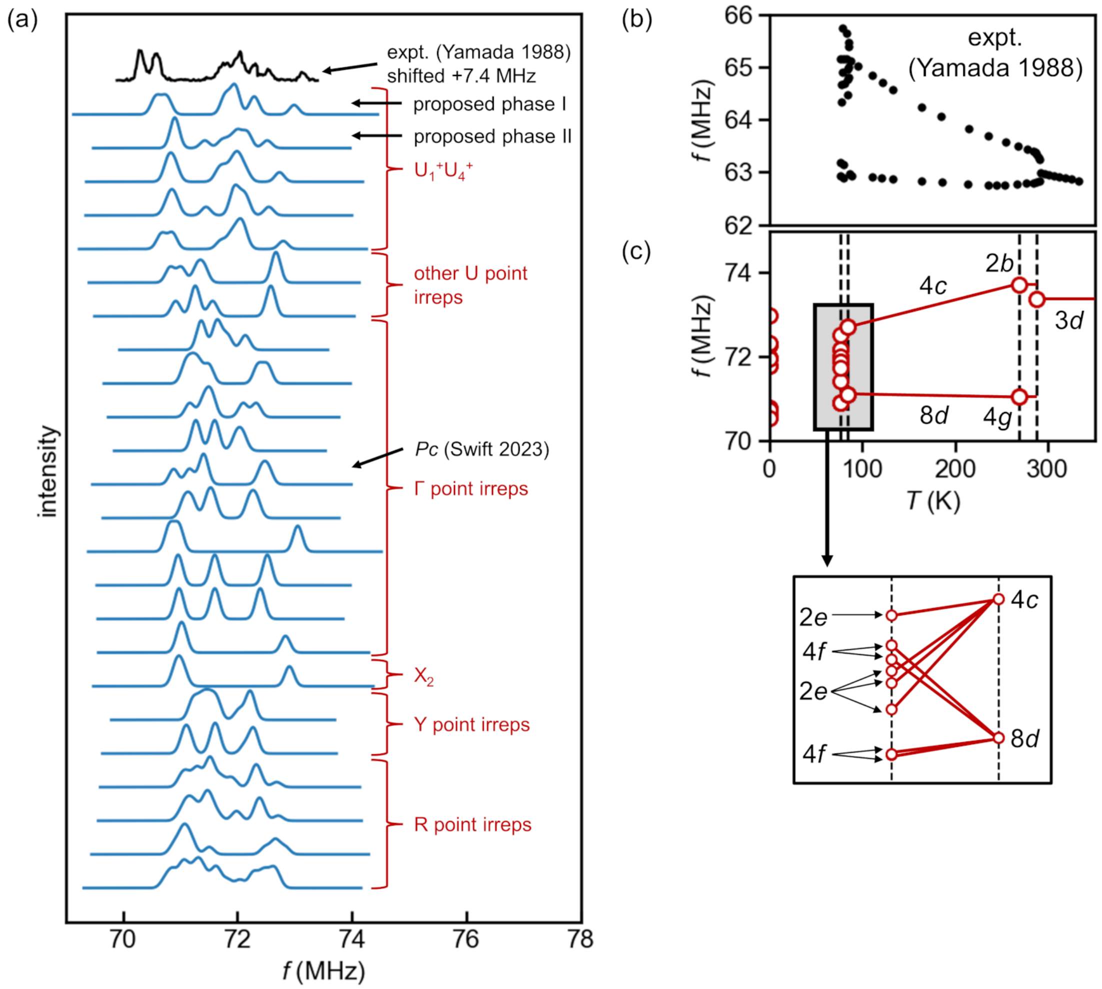

Figure S12: (a) Experimental ^{81}Br NQR spin echo-detected spectrum at 77 K reported by Yamada *et al.*[24] and computed spectra (PBE, arbitrary Gaussian broadening of central transition frequencies) for the candidate structures from first-principles phonon mode mapping. When accounting for a frequency shift (which captures both systematic errors in the computed electric field gradients and possible errors in the tabulated quadrupole moment, which continue to be refined over time),[25] there is strong resemblance between experiment and the spectrum computed for the proposed structure of phase I. The computed spectrum for the proposed structure of phase II is less similar, suggesting order–disorder character to the phase I–II transition. Other candidates, including the recently proposed Pc model of Swift and Lyons,[26] are seen not to reproduce the observed spectrum. The irreducible representations ("irreps") of the first distortions from phase III are annotated in red for the theoretical spectra. (b) Experimental temperature-dependence of ^{81}Br NQR frequencies reported by Yamada *et al.*[24] (c) Athermal ^{81}Br NQR frequencies computed for phases I–V. The Br Wyckoff sites and their splittings are annotated, and phase transition temperatures are marked with dashed lines. Data from Yamada *et al.* reproduced or adapted with permission from Ref. 24. Copyright 1988 Chemical Society of Japan.

9 Synchrotron powder diffraction

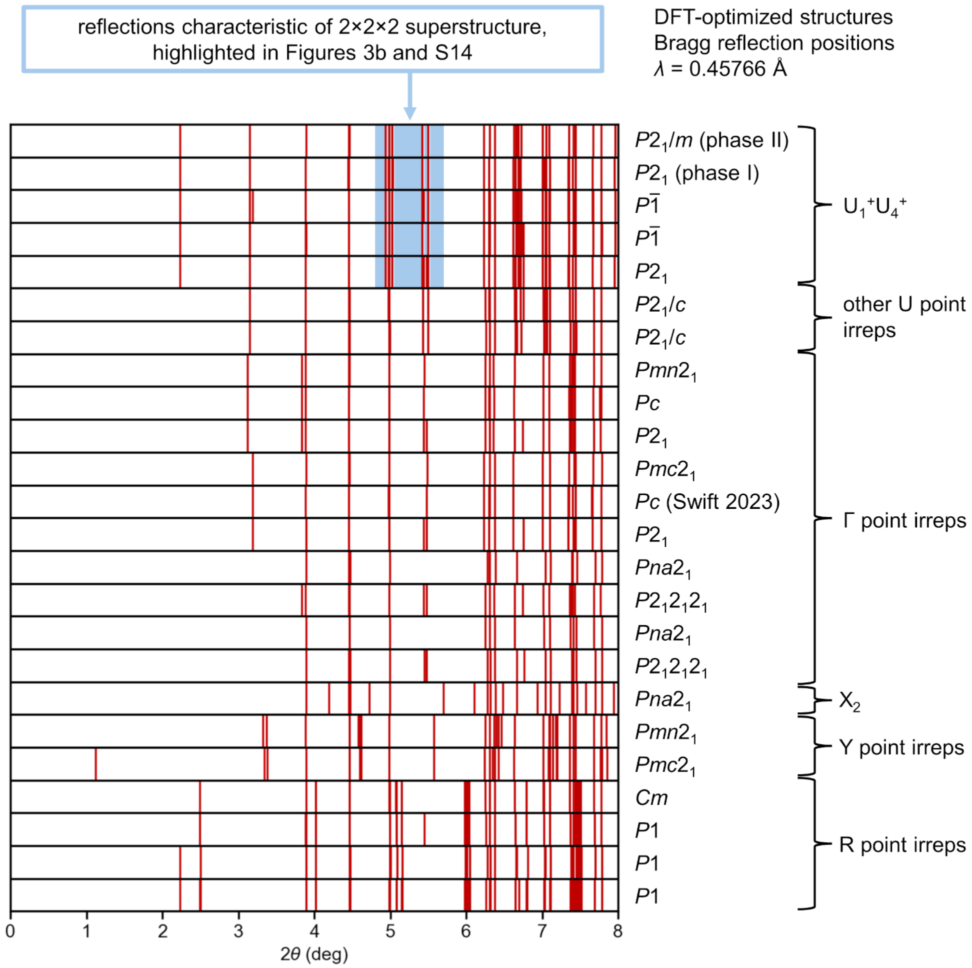

Figure S13: Bragg reflection angles from DFT-optimized candidate structure models from first-principles phonon mode mapping, which include structures proposed by others ("Swift 2023")[26] and those with precedent among reported perovskites (the first $Pmn2_1$, $Pmc2_1$, and $Pna2_1$ phases in the list). The space group of each structure and the irreducible representations ("irreps") of the first distortions from phase III are annotated. Only those structures which involve a distortion with irrep $U_1^+U_4^+$, which results in a 2×2×2 superstructure with respect to the cubic aristotype, lead to Bragg reflections consistent with experiment, as highlighted in the characteristic region around $2\theta \approx 5^\circ$ (see Figures 3b and S13). These five $U_1^+U_4^+$ phases include the proposed structures of phases I and II. The $U_1^+U_4^+$ phases with $P\bar{1}$ space group symmetry are higher in energy in DFT and are incompatible with the measured SHG and the clearly observed systematic absences from the 2_1 screw axis. The last $U_1^+U_4^+$ phase with $P2_1$ symmetry, the ferrielectric analog of the proposed structure of phase I, is higher in energy in DFT and does not lead to the pronounced elongation of the b axis observed by diffraction.

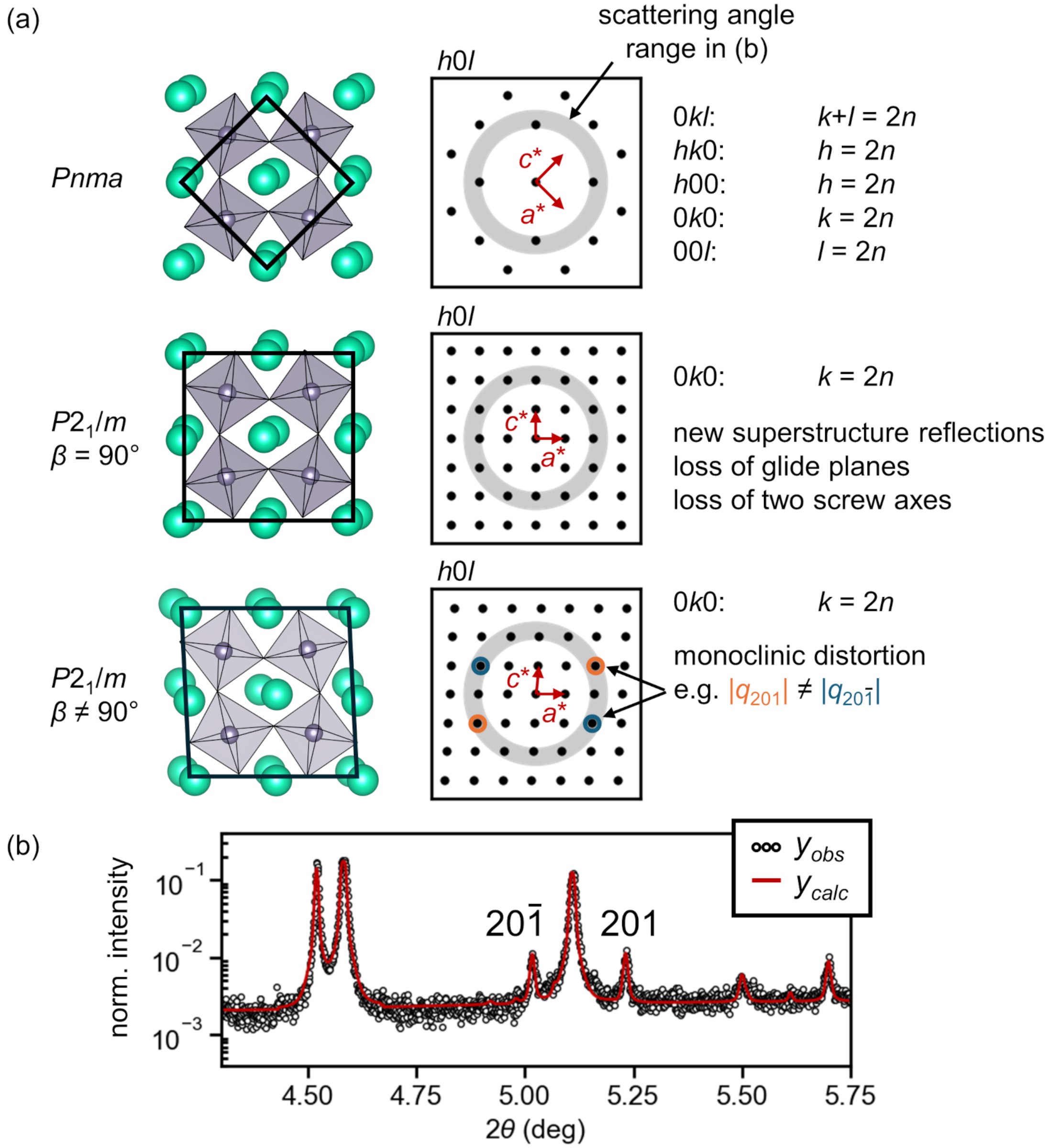

Figure S14: Evolution of Bragg reflections in reciprocal space on formation of the superstructure of phases I and II. (a) Real space structures, Bragg reflections in the $h0l$ plane, and systematic absence conditions for phase III ($Pnma$) and phases I and II ($P2_1$ and $P2_1/m$, same Friedel symmetry). The changes are separated into two steps: 1) Superstructuring, which enlarges the unit cell and breaks several screw axis and glide plane symmetries but maintains a metrically orthorhombic cell, and 2) monoclinic distortion, which leads to Bragg peak splitting. The grey annuli are the intersection of the spherical annulus captured by the scattering angles in (b) with the $h0l$ plane. (b) Excerpt of the graphical result of Rietveld refinement of powder diffraction at 7.9 K in space group $P2_1$, showing several key superstructure reflections. “norm. intensity” = scattered intensity, normalized to the highest Bragg reflection.

Table S3: Crystallographic data of $CsSnBr_3$ phase I refined using space groups $P2_1/m$ and $P2_1$. (*) As defined in TOPAS (Bruker AXS).

sum formula	$CsSnBr_3$	
molecular weight (g mol^{-1})	491.327	
temperature (K)	7.9 K	
space group	$P2_1/m$ (#11)	$P2_1$ (#4)
Z	8	8
a (Å)	11.45578(3)	11.45580(3)
b (Å)	11.60566(2)	11.60565(2)
c (Å)	11.47295(3)	11.47298(3)
β (°)	93.0070(3)	93.0066(3)
V (Å^3)	1523.250(7)	1523.25(1)
ρ_{calc} (g cm^{-3})	4.28	4.28
wavelength (Å)	0.45766	
R_p (%)*	10.7	10.6
R_{wp} (%)*	14.1	13.9
R_{F^2} (%)*	5.5	5.4
R_{exp} (%)*	6.8	6.8
G.O.F.*	2.06	2.04
no. of variables	90	116

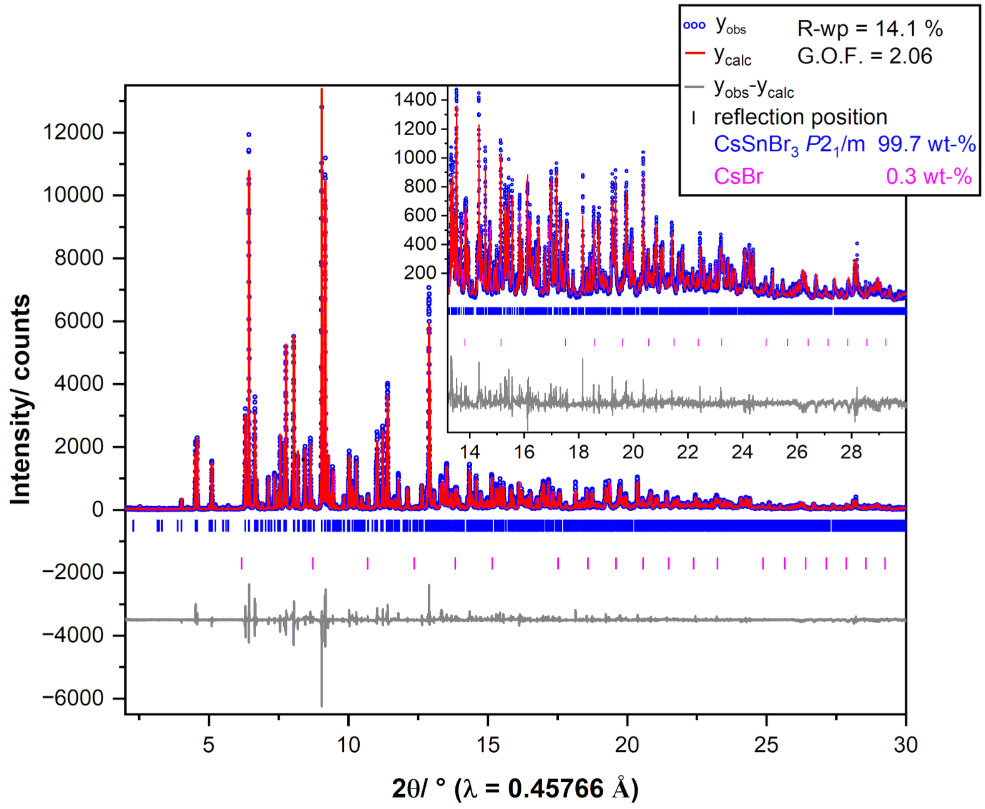

Figure S15: Graphical result of the Rietveld refinement of $CsSnBr_3$ PXRD at 7.9 K using space group $P2_1/m$. The intensity scale in the high angle region in the inset is enlarged by a factor of 9 for clarity. Analogous data for refinement using space group $P2_1$ are presented in Figure 3d.

Table S4: Atomic coordinates of $CsSnBr_3$ phase I refined using space groups $P2_1/m$ and $P2_1$ at 7.9 K. (*) Isotropic atomic displacement parameters were constrained to be equivalent across distinct sites for each element. "Wyck." = Wyckoff position, "Site" = site symmetry, "S.O.F." = site occupation factor.

**Atom**	**Wyck.**	**Site**	**S.O.F.**	**x / a**	**y / b**	**z / c**	**B (Å^2)**
				$P2_1/m$ model			
Cs1	2e	m	1	0.5504(3)	1/4	−0.0240(3)	0.73(2)*
Cs2	2e	m	1	0.0284(3)	1/4	0.4820(3)	0.73(2)*
Cs3	2e	m	1	0.5139(3)	1/4	0.4530(3)	0.73(2)*
Cs4	2e	m	1	0.0267(3)	1/4	−0.0341(3)	0.73(2)*
Sn1	4f	1	1	0.7561(3)	0.4978(3)	0.7455(3)	0.49(2)*
Sn2	4f	1	1	0.7455(3)	−0.0007(4)	0.2436(3)	0.49(2)*
Br1	4f	1	1	0.0090(3)	0.5288(5)	0.2978(3)	0.80(2)*
Br2	4f	1	1	0.4956(3)	0.5316(4)	0.8048(3)	0.80(2)*
Br3	4f	1	1	0.7016(3)	−0.0282(4)	0.4823(3)	0.80(2)*
Br4	4f	1	1	0.1987(3)	−0.0341(4)	0.0145(3)	0.80(2)*
Br5	2e	m	1	0.2208(5)	1/4	0.2382(4)	0.80(2)*
Br6	2e	m	1	0.7191(5)	1/4	0.7101(4)	0.80(2)*
Br7	2e	m	1	0.7730(5)	1/4	0.2730(5)	0.80(2)*
Br8	2e	m	1	0.2801(5)	1/4	0.7863(5)	0.80(2)*
				$P2_1$ model			
Cs1	2a	1	1	0.5497(3)	0.2552(8)	−0.0243(3)	0.75(3)*
Cs2	2a	1	1	0.0294(3)	0.2499(9)	0.4824(3)	0.75(3)*
Cs3	2a	1	1	0.5142(3)	0.2484(11)	0.4530(3)	0.75(3)*
Cs4	2a	1	1	0.0266(3)	0.2507(11)	−0.0337(3)	0.75(3)*
Sn1a	2a	1	1	0.7525(6)	0.4970(6)	0.7524(6)	0.44(3)*
Sn1b	2a	1	1	0.2410(6)	0.5006(5)	0.2610(6)	0.44(3)*
Sn2a	2a	1	1	0.7443(7)	0.0008(6)	0.2430(6)	0.44(3)*
Sn2b	2a	1	1	0.2551(7)	0.0022(6)	0.7554(6)	0.44(3)*
Br1a	2a	1	1	0.0084(9)	0.5258(10)	0.2958(8)	0.60(2)*
Br1b	2a	1	1	0.9910(9)	0.4690(10)	0.7000(9)	0.60(2)*
Br2a	2a	1	1	0.4869(8)	0.5313(9)	0.8095(7)	0.60(2)*
Br2b	2a	1	1	0.4962(8)	0.4663(9)	0.2005(8)	0.60(2)*
Br3a	2a	1	1	0.7062(8)	−0.0248(9)	0.4859(8)	0.60(2)*
Br3b	2a	1	1	0.3039(8)	0.0304(9)	0.5210(8)	0.60(2)*
Br4a	2a	1	1	0.1990(9)	−0.0307(9)	0.0085(8)	0.60(2)*
Br4b	2a	1	1	0.8019(8)	0.0373(9)	0.9799(8)	0.60(2)*
Br5	2a	1	1	0.2214(5)	0.2335(7)	0.2382(4)	0.60(2)*
Br6	2a	1	1	0.7191(5)	0.2431(10)	0.7109(4)	0.60(2)*
Br7	2a	1	1	0.7730(5)	0.2426(12)	0.2735(5)	0.60(2)*
Br8	2a	1	1	0.2803(5)	0.2408(11)	0.7858(5)	0.60(2)*

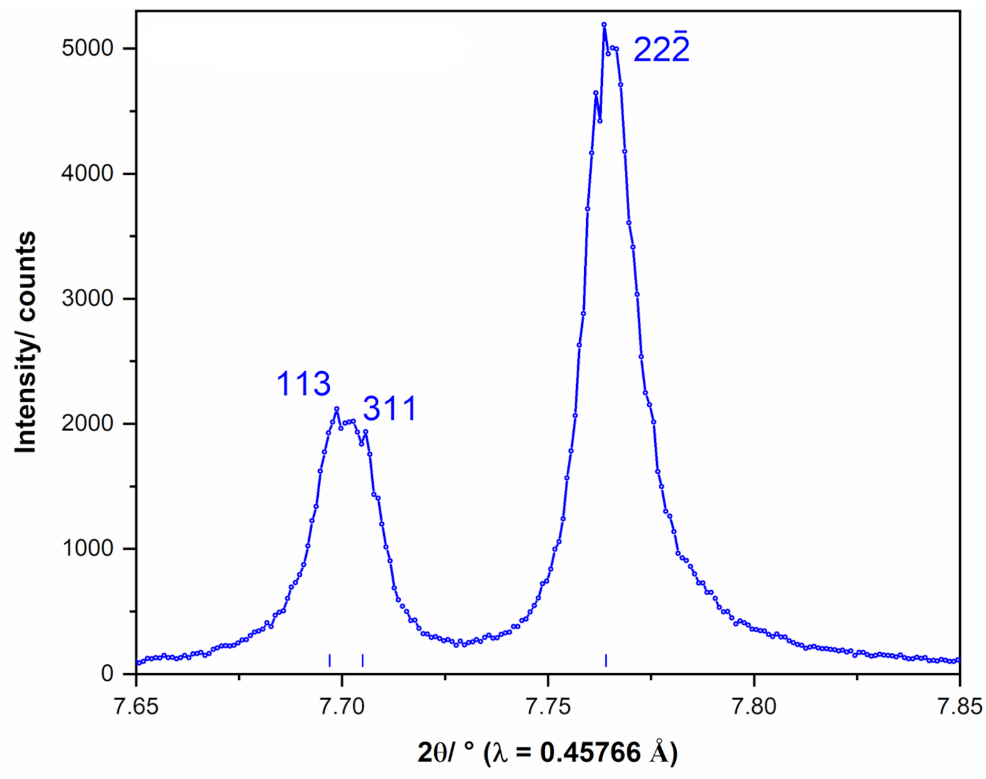

Figure S16: Excerpt of the PXRD pattern of $CsSnBr_3$ recorded at 7.9 K, with reflection indices annotated.

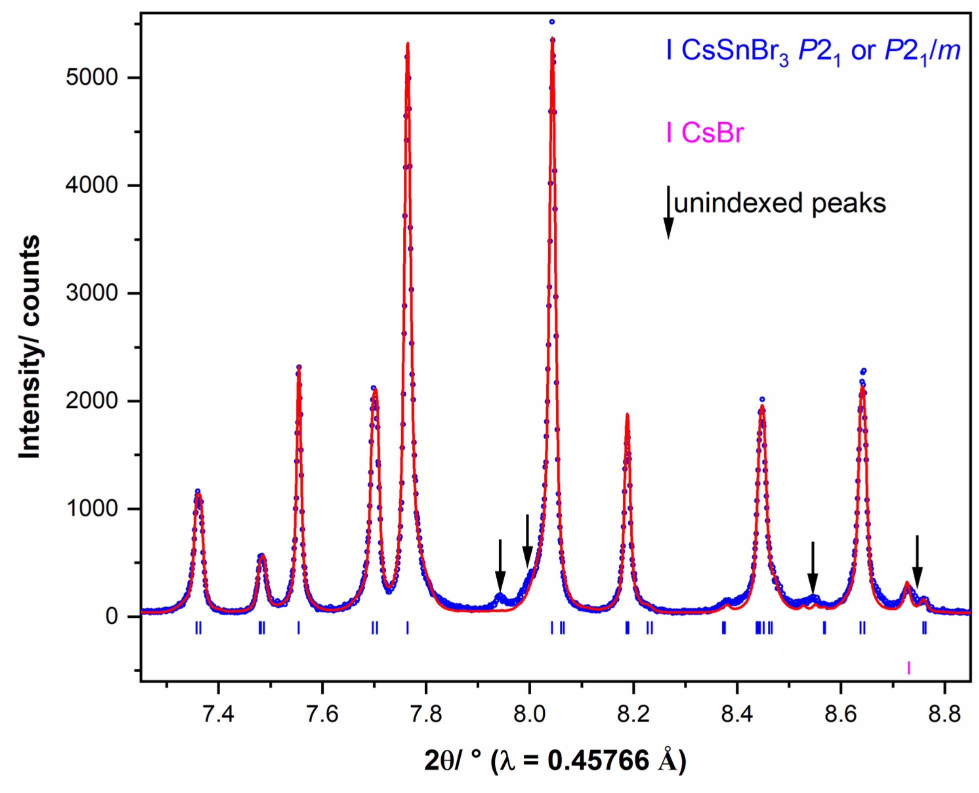

Figure S17: Excerpt of the graphical result of the refinement of $CsSnBr_3$ PXRD at 7.9 K using space group $P2_1/m$. The black arrows indicate minor, unindexed peaks.

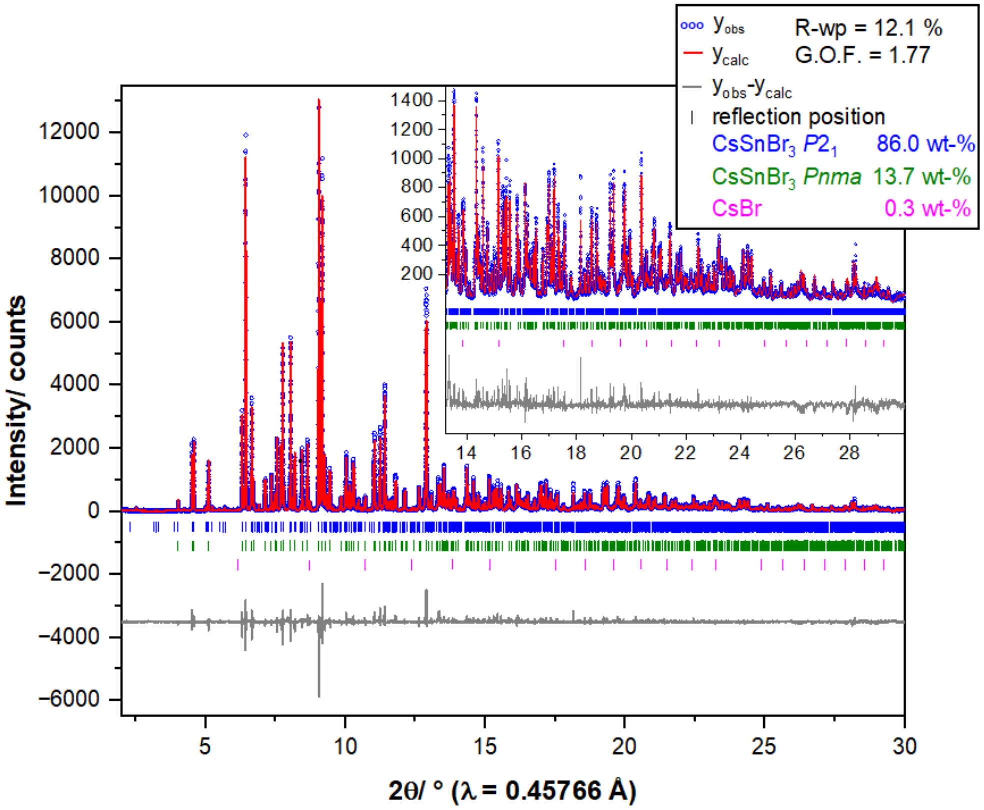

Figure S18: Graphical result of the Rietveld refinement of $CsSnBr_3$ PXRD at 7.9 K using space group $P2_1$, with a second phase of $Pnma$ symmetry included (see main text). The intensity scale in the high angle region in the inset is enlarged by a factor of 9 for clarity.

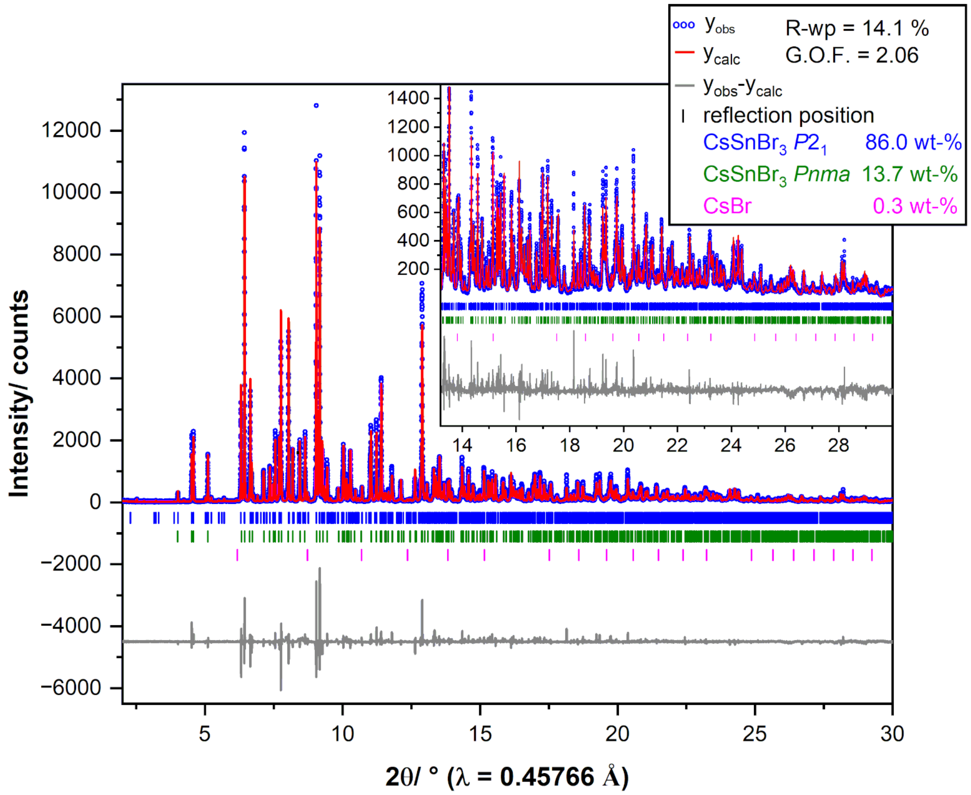

Figure S19: Graphical result of the Rietveld refinement of $CsSnBr_3$ PXRD at 7.9 K using space group $P2_1$, with a second phase of $Pnma$ symmetry included, without the inclusion of symmetry adapted spherical harmonics or the Stephens model (see main text). The intensity scale in the high angle region in the inset is enlarged by a factor of 9 for clarity.

10 Polarization−orientation-dependence of Raman scattering from birefringent crystals in point group 2

The Raman tensors for the two distinct symmetries (A and B) of modes possible for crystals in point group 2 are

$$R_A = \begin{pmatrix} a_{11} & 0 & a_{13} \\ 0 & a_{22} & 0 \\ a_{13} & 0 & a_{33} \end{pmatrix}, \tag{1}$$

and

$$R_B = \begin{pmatrix} 0 & b_{12} & 0 \\ b_{12} & 0 & b_{23} \\ 0 & b_{23} & 0 \end{pmatrix}. \tag{2}$$

The intensity of Raman scattering is taken to be:[27]

$$I \propto \left| \hat{e_s} J R J \hat{e_i}^T \right|^2, \tag{3}$$

where $\hat{e_i}$ and $\hat{e_s}$ are the Jones vectors for the electric field of the (linearly polarized) incident and scattered light, respectively, and J is a Jones matrix accounting for sample linear birefringence, expressed as:

$$J = \begin{pmatrix} 1 & 0 & 0 \\ 0 & e^{i\phi_y} & 0 \\ 0 & 0 & e^{i\phi_z} \end{pmatrix}. \tag{4}$$

Circular birefringence of the sample is not included despite its chiral symmetry because optical rotation cancels along the incident and scattered paths in backscattering geometry.[28] Because the $CsSnBr_3$ crystals grow with $\{100\}$ faceting in the cubic phase, rather than treat sample orientation by an explicit rotation matrix relating the lab frame to the crystal frame, we simply consider light along the same x, y, and z Cartesian axes with which we express the Raman tensors and then allow for an extra offset angle, θ_0, in the argument of every trigonometric function expressing rotation of the sample around the light propagation direction (*i.e.*, $\hat{e}(\theta) \to \hat{e}(\theta + \theta_0)$). During fitting, we also allow for slight misalignment, δ, between incident and scattered beam polarizers (*i.e.*, $\hat{e_s}(\theta + \theta_0) \to \hat{e_s}(\theta + \theta_0 + \delta)$). ϕ_y, ϕ_z, θ_0, and δ are the same across all observed Raman modes in the same experiment, so a simultaneous fitting procedure is employed. The electric field Jones vectors for incident and scattered light are given in Table S5. With this form of the Raman tensors, the monoclinic lattice vectors in the Cartesian basis are $\vec{a} = (a, 0, 0)$, $\vec{b} = (0, b, 0)$, and $\vec{c} = (c\cos\beta, 0, c\sin\beta)$, so $\vec{b} \parallel \hat{y}$.

Table S5: Jones vectors for incident ($\hat{e_i}$) and scattered light in parallel ($\hat{e}_{s,\parallel}$) and cross ($\hat{e}_{s,\perp}$) polarization.

	light along $\hat{x}$	light along $\hat{y}$	light along $\hat{z}$
$\hat{e_i}$	$(0, \cos\theta, \sin\theta)$	$(\sin\theta, 0, \cos\theta)$	$(\cos\theta, \sin\theta, 0)$
$\hat{e}_{s,\parallel}$	$(0, \cos\theta, \sin\theta)$	$(\sin\theta, 0, \cos\theta)$	$(\cos\theta, \sin\theta, 0)$
$\hat{e}_{s,\perp}$	$(0, -\sin\theta, \cos\theta)$	$(\cos\theta, 0, -\sin\theta)$	$(-\sin\theta, \cos\theta, 0)$

This leads to the expressions in Tables S6 and S7 for Raman modes of A and B symmetry, respectively, for each direction of light propagation with respect to the pseudocubic axes and each polarization configuration (parallel- or cross-polarized incident and scattered beams) for the case $\theta_0 = \delta = 0$.

Table S6: Raman intensity sub-expression, $\hat{e_s} J R_A J \hat{e_i}^T$, for mode symmetry A for various light propagation directions in the crystal frame of reference.

light direction	polarization	$\hat{e_s} J R_A J \hat{e_i}^T$
$\hat{x}$	$\parallel$	$\frac{1}{2}\left(a_{22}\, e^{2i\phi_y} + a_{33}\, e^{2i\phi_z}\right) + \frac{1}{2}\left(a_{22}\, e^{2i\phi_y} - a_{33}\, e^{2i\phi_z}\right)\cos 2\theta$
$\hat{x}$	$\perp$	$\frac{1}{2}\left(a_{33} e^{2i\phi_z} - a_{22} e^{2i\phi_y}\right)\sin 2\theta$
$\hat{y}$	$\parallel$	$\frac{1}{2}\left(a_{11} + a_{33} e^{2i\phi_z}\right) + a_{13}\, e^{i\phi_z}\sin 2\theta + \frac{1}{2}\left(a_{33}\, e^{2i\phi_z} - a_{11}\right)\cos 2\theta$
$\hat{y}$	$\perp$	$\frac{1}{2}\left(a_{11} - a_{33}\, e^{2i\phi_z}\right)\sin 2\theta + a_{13}\, e^{i\phi_z}\cos 2\theta$
$\hat{z}$	$\parallel$	$\frac{1}{2}\left(a_{11} + a_{22}\, e^{2i\phi_y}\right) + \frac{1}{2}\left(a_{11} - a_{22}\, e^{2i\phi_y}\right)\cos 2\theta$
$\hat{z}$	$\perp$	$\frac{1}{2}\left(a_{22}\, e^{2i\phi_y} - a_{11}\right)\sin 2\theta$

Table S7: Raman intensity sub-expression, $\hat{e_s} J R_B J \hat{e_i}^T$, for mode symmetry B for various light propagation directions in the crystal frame of reference. Notably, modes of B symmetry are silent when the monoclinic b axis is parallel to the incident and scattered beams.

light direction	polarization	$\hat{e_s} J R_B J \hat{e_i}^T$
$\hat{x}$	$\parallel$	$b_{23}\, e^{i(\phi_y+\phi_z)} \sin 2\theta$
$\hat{x}$	$\perp$	$b_{23}\, e^{i(\phi_y+\phi_z)} \cos 2\theta$
$\hat{y}$	$\parallel$	0
$\hat{y}$	$\perp$	0
$\hat{z}$	$\parallel$	$b_{12}\, e^{i\phi_y} \sin 2\theta$
$\hat{z}$	$\perp$	$b_{12}\, e^{i\phi_y} \cos 2\theta$

Given the three second-order phase transitions on cooling, we consider also the possibility of microscopic transformation twins. We write the intensity of Raman scattering from a microtwinned crystal, including linear birefringence, as

$$I \propto \left| \hat{e_s} J \left[fTR + (1-f)R \right] J \hat{e_i}^T \right|^2 , \tag{5}$$

where T is the linear transformation describing the twin operation and f is the volume fraction of the twinned domains (taken to be $\frac{1}{2}$ in the absence of external stimuli which could bias the relative domain populations). We consider 16 possible transformation twins, summarized in Table S8: 8 are symmetry elements lost in the $P4/mbm \rightarrow Pnma$ transition, 4 are symmetry elements lost in the $Pnma \rightarrow P2_1/m$ transition, 2 are symmetry elements lost in the $P2_1/m \rightarrow P2_1$ transition, and 2 are the elements which remain in the $P2_1$ phase but which we include so that the considered operations form a group – as such, twins formed by multiple operations are implicitly included as the product of any two elements of the group is another element of the group.

Considering these 16 possible twin operations for 3 distinct directions of light propagation with respect to the pseudocubic axes and for 2 different Raman mode symmetries leads to 96 distinct scenarios, each with 2 relative polarizations of incident and scattered beams (for a total of 192 cases). Due to this large number of scenarios, we numerically compute the scattering for arbitrary Raman tensor elements and screen for microtwinning−orientation−mode symmetry scenarios which render a mode silent. We find that 6 scenarios cause A modes to be silent, and 12 scenarios cause B modes to be silent. However, all 6 scenarios which produce silent A modes also produce silent B modes (these include inversion twinning) and are thus incompatible with our observations. The remaining 6 scenarios, for which A modes are observed but B modes are silent, are all for light propagation along $\hat{y}$, *i.e.* they are qualitatively identical to the untwinned case. For quantitative comparison, we performed the same simultaneous fitting procedure of parallel- and cross-polarized scattering data as described in the main text for each of these 6 different twin operations with light propagation along $\hat{y}$: E (corresponding to the untwinned case), $m \perp \hat{y}$, $2 \parallel \hat{x}$, $m \perp \hat{x}$, $2 \parallel \hat{z}$, and $m \perp \hat{z}$. We find that $m \perp \hat{y}$ twins, *i.e.* reflection twins formed during the $P2_1/m \rightarrow P2_1$ transition, lead to the exact same functional form for the PO-dependence as the untwinned case. Therefore, neither can we detect their possible presence nor would they alter the conclusions of our symmetry analysis. On the other hand, the 4 other twins which produce silent B modes and observable A modes (the mirrors and 2-fold rotations lost in the $Pnma \rightarrow P2_1/m$ transition) are incompatible with the observed scattering, with substantially worse fits for these twinned models than for the untwinned one. Therefore, we can conclude that twinning does not account for the silence of B modes in the experiments, which instead appears to be due to the macroscopic orientation of the crystals, which is set by the uncontrolled nucleation of the tetragonal phase on cooling through the first order cubic−tetragonal transition. Additionally, no twinning is necessary to explain our observations, though there could be undetectable reflection twins associated with the loss of the mirror plane from $P2_1/m$ to $P2_1$.

Table S8: Symmetry operations considered as possible transformation twins in the second-order transitions from $P4/mbm \rightarrow P2_1$.

point group operation	T	notes
E	$\begin{pmatrix}1&0&0\\0&1&0\\0&0&1\end{pmatrix}$	retained in point group 2
$2 \parallel \hat{y}$	$\begin{pmatrix}-1&0&0\\0&1&0\\0&0&-1\end{pmatrix}$	retained in point group 2
$m \perp \hat{y}$	$\begin{pmatrix}1&0&0\\0&-1&0\\0&0&1\end{pmatrix}$	lost from $2/m \rightarrow 2$
i	$\begin{pmatrix}-1&0&0\\0&-1&0\\0&0&-1\end{pmatrix}$	lost from $2/m \rightarrow 2$
$2 \parallel \hat{x}$	$\begin{pmatrix}1&0&0\\0&-1&0\\0&0&-1\end{pmatrix}$	lost from $\frac{2}{m}\frac{2}{m}\frac{2}{m} \rightarrow 2/m$
$m \perp \hat{x}$	$\begin{pmatrix}-1&0&0\\0&1&0\\0&0&1\end{pmatrix}$	lost from $\frac{2}{m}\frac{2}{m}\frac{2}{m} \rightarrow 2/m$
$2 \parallel \hat{z}$	$\begin{pmatrix}-1&0&0\\0&-1&0\\0&0&1\end{pmatrix}$	lost from $\frac{2}{m}\frac{2}{m}\frac{2}{m} \rightarrow 2/m$
$m \perp \hat{z}$	$\begin{pmatrix}1&0&0\\0&1&0\\0&0&-1\end{pmatrix}$	lost from $\frac{2}{m}\frac{2}{m}\frac{2}{m} \rightarrow 2/m$
$4 \parallel \hat{z}$	$\begin{pmatrix}0&-1&0\\1&0&0\\0&0&1\end{pmatrix}$	lost from $\frac{4}{m}\frac{2}{m}\frac{2}{m} \rightarrow \frac{2}{m}\frac{2}{m}\frac{2}{m}$
$4^{-1} \parallel \hat{z}$	$\begin{pmatrix}0&1&0\\-1&0&0\\0&0&1\end{pmatrix}$	lost from $\frac{4}{m}\frac{2}{m}\frac{2}{m} \rightarrow \frac{2}{m}\frac{2}{m}\frac{2}{m}$
$\bar{4} \parallel \hat{z}$	$\begin{pmatrix}0&1&0\\-1&0&0\\0&0&-1\end{pmatrix}$	lost from $\frac{4}{m}\frac{2}{m}\frac{2}{m} \rightarrow \frac{2}{m}\frac{2}{m}\frac{2}{m}$
$\bar{4}^{-1} \parallel \hat{z}$	$\begin{pmatrix}0&-1&0\\1&0&0\\0&0&-1\end{pmatrix}$	lost from $\frac{4}{m}\frac{2}{m}\frac{2}{m} \rightarrow \frac{2}{m}\frac{2}{m}\frac{2}{m}$
$2 \parallel (\hat{x}+\hat{y})$	$\begin{pmatrix}0&1&0\\1&0&0\\0&0&-1\end{pmatrix}$	lost from $\frac{4}{m}\frac{2}{m}\frac{2}{m} \rightarrow \frac{2}{m}\frac{2}{m}\frac{2}{m}$
$2 \parallel (\hat{y}-\hat{x})$	$\begin{pmatrix}0&-1&0\\-1&0&0\\0&0&-1\end{pmatrix}$	lost from $\frac{4}{m}\frac{2}{m}\frac{2}{m} \rightarrow \frac{2}{m}\frac{2}{m}\frac{2}{m}$
$\bar{2} \parallel (\hat{x}+\hat{y})$	$\begin{pmatrix}0&-1&0\\-1&0&0\\0&0&1\end{pmatrix}$	lost from $\frac{4}{m}\frac{2}{m}\frac{2}{m} \rightarrow \frac{2}{m}\frac{2}{m}\frac{2}{m}$
$\bar{2} \parallel (\hat{y}-\hat{x})$	$\begin{pmatrix}0&1&0\\1&0&0\\0&0&1\end{pmatrix}$	lost from $\frac{4}{m}\frac{2}{m}\frac{2}{m} \rightarrow \frac{2}{m}\frac{2}{m}\frac{2}{m}$

11 Raman spectra and analysis

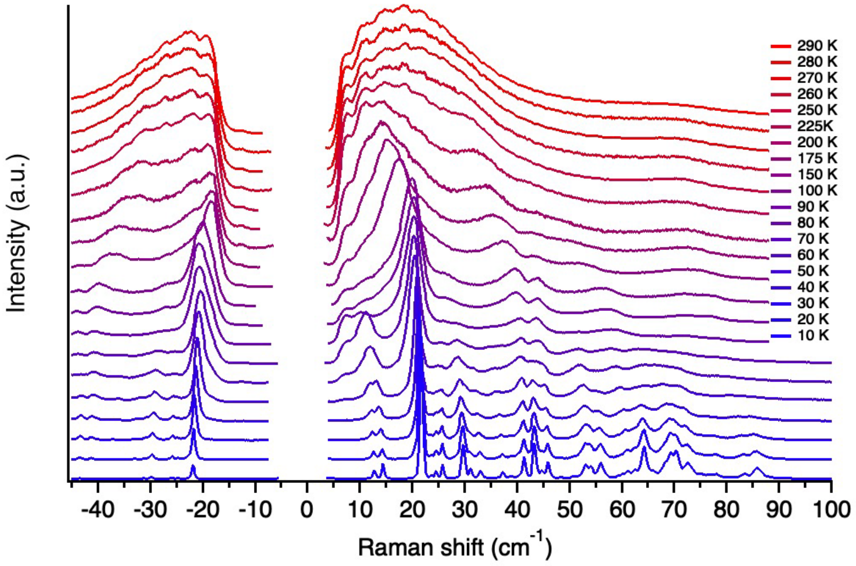

Figure S20: Unpolarized Raman spectra for $CsSnBr_3$ up to 100 cm^{-1} Stokes shift at temperatures between 10 K and ambient.

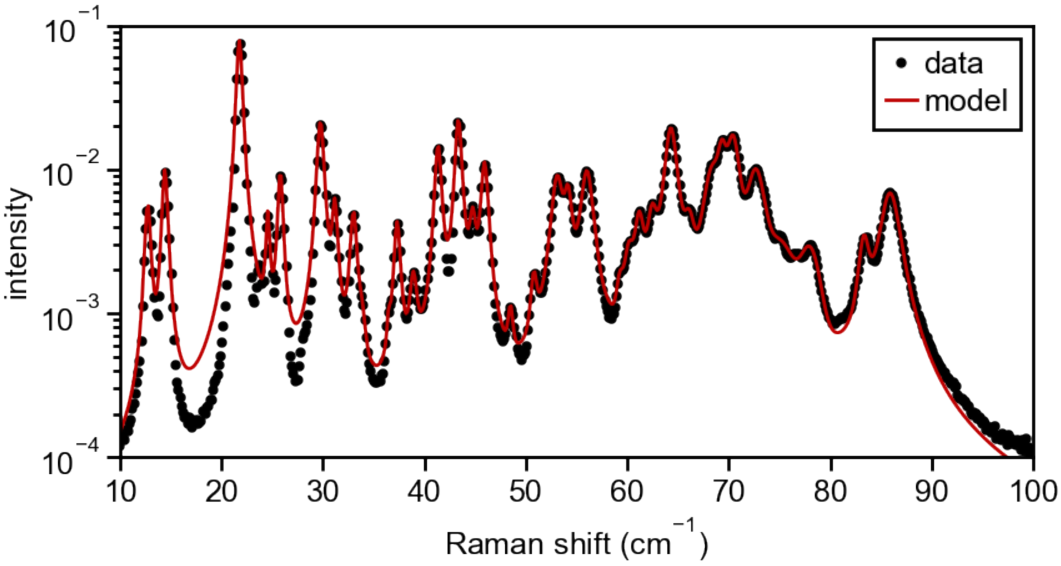

Figure S21: Unpolarized Raman spectrum at 10 K up to 100 cm^{-1} Stokes shift, fit to a sum of 36 pseudo-Voigt peaks. Additional weak modes which overlap the shoulders of strong peaks refine to zero intensity due to the imperfect peak profile, suggesting 36 is a lower bound on the true number of modes. See main text.

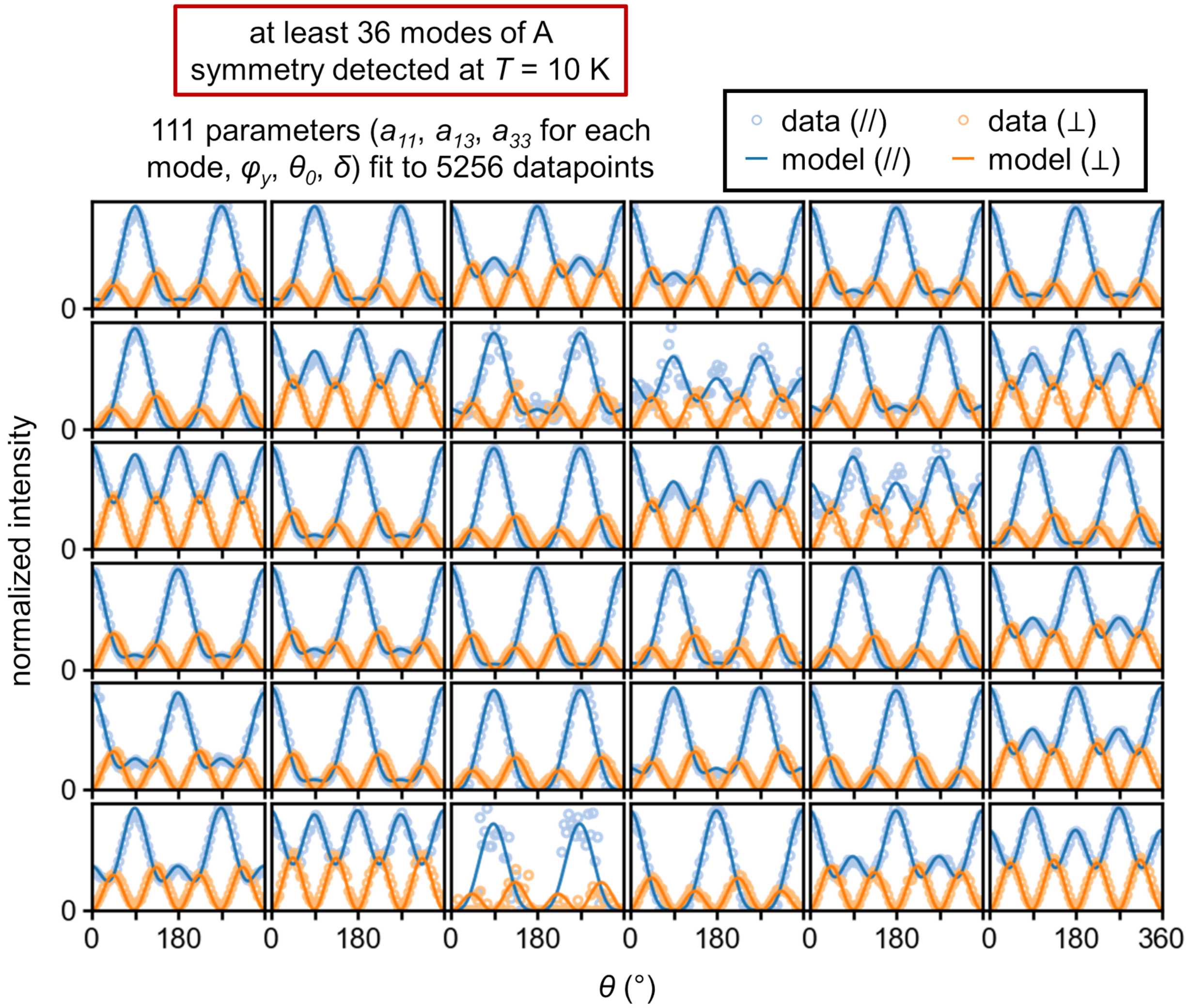

Figure S22: Fitting of the polarization-orientation dependence of Raman scattering from a $CsSnBr_3$ single crystal at T = 10 K (phase I, space group $P2_1$), including the effects of optical birefringence. The intensities of at least 36 modes within the measurement window (Stokes shift $<$ 100 cm^{-1}) can be robustly extracted, with manual inspection of the spectra suggesting more modes which are unresolved in frequency or very weak in intensity. All 36 resolved modes are well fit by A symmetry. Solid lines are fits to Equation 3. Similar results are observed for two other crystals. No modes of B symmetry are observed in the measured crystals, which is consistent with light propagation parallel to the b axis (see Table S7 and discussion in main text).

Table S9: Factor group analysis of the proposed structures of $CsSnBr_3$ and alternative models (either from our first-principles calculations, proposed structures reported by others,[26] or enumerated possibilities arising from combinations of tilting[9] and ferroic distortions[4]). Only the proposed structure of phase I is supported by the PO Raman experiments at 10 K (see main text). All alternative models are incompatible with the number of Raman modes and their symmetries, and are additionally disproven by X-ray diffraction: Orthorhombic phases do not account for the observed monoclinic distortion, and none of the monoclinic alternatives account for the observed superstructure reflections associated with the noncollinear distortions enlarging the unit cell in the ac-plane. Note that the two phases with space group symmetry Pc are distinct structures with different unit cells despite sharing the same Wyckoff sequence and Pearson symbol.

Space group	Pearson symbol	Wyckoff sequence	Phase	Raman-active modes
$Pnma$	$oP20$	dc^2a	**phase III**	$7A_g+5B_{1g}+7B_{2g}+5B_{3g}$
$P2_1/m$	$mP40$	f^6e^8	**proposed phase II**	$34A_g+26B_g$
$P2_1$	$mP40$	a^{20}	**proposed phase I**	60A+60B (acoustic: A+2B)
$P2_1/m$	$mP20$	f^2e^4da	"Ref 14" in Stokes *et al.*,[4] center of bottom row in Figure 2 of Bock & Müller[9]	$14A_g+10B_g$
$Pmn2_1$	$oP20$	b^3a^4	#1 in Figure 2, "Ref 58" in Stokes *et al.*[4]	$17A_1+13A_2+13B_1+17B_2$ (acoustic: $A_1+B_1+B_2$)
$Pmc2_1$	$oP20$	$c^3b^2a^2$	#2 in Figure 2, "Ref 57" in Stokes *et al.*[4]	$17A_1+13A_2+13B_1+17B_2$ (acoustic: $A_1+B_1+B_2$)
$Pna2_1$	$oP20$	a^5	#4 in Figure 2, "Ref 56" in Stokes *et al.*[4]	$15A_1+15A_2+15B_1+15B_2$ (acoustic: $A_1+B_1+B_2$)
Pc	$mP20$	a^{10}	#1 in Figure S6, "Ref 59" in Stokes *et al.*[4]	$30A'+30A''$ (acoustic: $2A'+A''$)
Pc	$mP20$	a^{10}	#3 in Figure 2, proposed ground state in Swift & Lyons[26]	$30A'+30A''$ (acoustic: $2A'+A''$)
$P2_12_12_1$	$mP20$	a^5	#5 in Figure 2	$15A+15B_1+15B_2+15B_3$ (acoustic: $B_1+B_2+B_3$)

12 Comparison of experimental and calculated structures

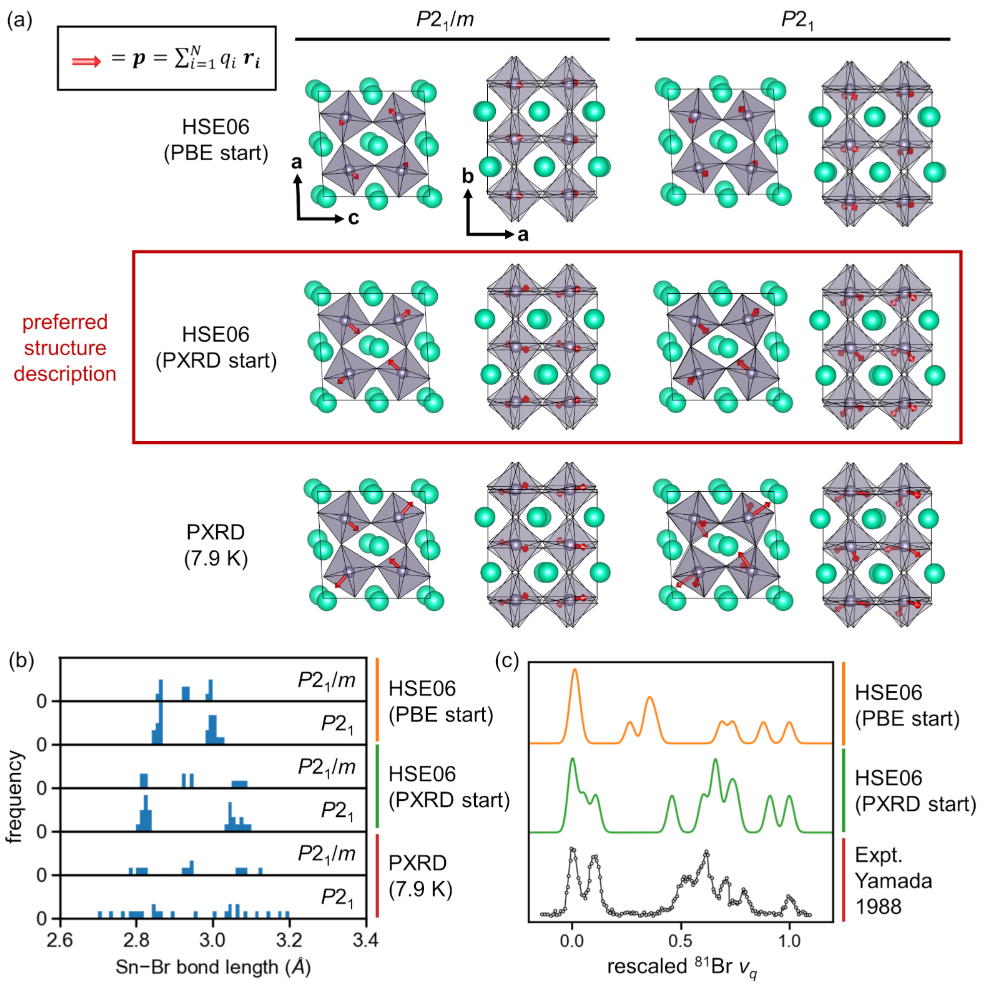

Figure S23: (a) Comparison of calculated and experimental structure models of $CsSnBr_3$ phases I and II. Atom positions are relaxed in the HSE06-calculated models, while the unit cell is fixed to that from synchrotron PXRD at 7.9 K. "PBE start" models are initiated from the atom positions of the DFT-only modemapping (PBE), while "PXRD start" models are initiated from the Rietveld-refined experimental structures. Though the two sets of models exhibit the same symmetries and result from distortions with the same irreducible representations, the direction of the lone pair dipoles is better accommodated by the precise tilting and *A*-site displacements in the "PXRD start" models than by those in the "PBE start models." (b) The $P2_1$ structures exhibit bifurcated bond lengths reminiscent of the pyramidal coordination found in $CsGeX_3$ [14] and $CsSnF_3$.[10] The experimental bond lengths agree better with those of the "PXRD start" models than with those of the "PBE start" models, particularly for $P2_1/m$, where PXRD-derived atom positions are more reliable as they do not rely on limited anomalous scattering. (c) Comparison of ^{81}Br quadrupolar resonance frequencies from HSE06 electric field gradients with the experimental 77 K spectrum[24] (here, rescaled by minimum and maximum frequencies to span the same range) also supports the "PXRD start" model over the "PBE start" model. Data from Yamada *et al.* reproduced or adapted with permission from Ref. 24. Copyright 1988 Chemical Society of Japan.

13 Strain impacts on lone pair distortions

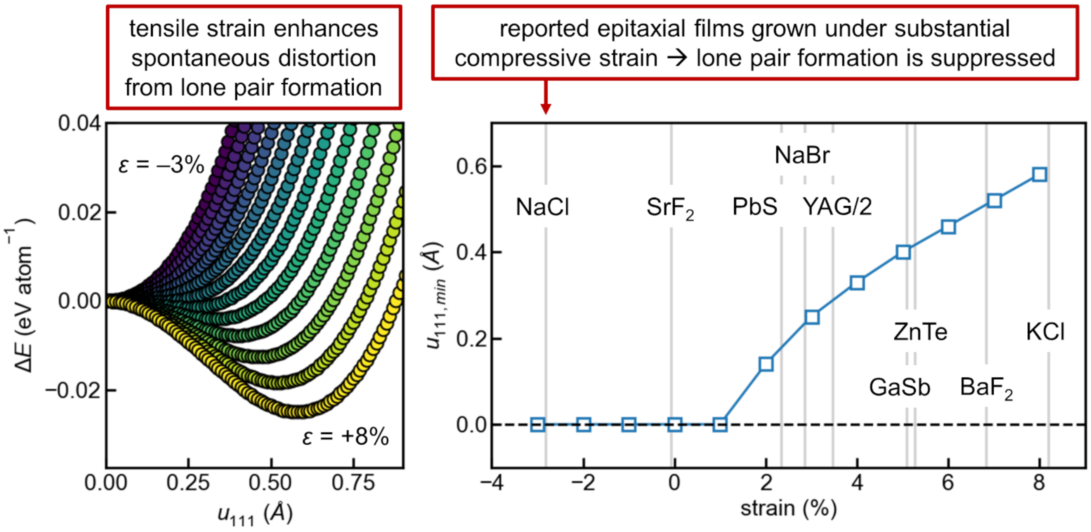

Figure S24: Qualitative impacts of strain on trigonal lone pair distortions in cubic $CsSnBr_3$. Left: DFT-computed total energies, ΔE, of trigonal Sn(II) displacements, u_{111}, for a range of compressive and tensile strains, ϵ. Right: Spontaneous trigonal displacement which minimizes the total energy, $u_{111,min}$, as a function of strain. The strains corresponding to various potential cubic substrates for epitaxial growth are indicated ("YAG/2" = half the lattice parameter for yttrium aluminum garnet). Notably, previously reported epitaxial $CsSnBr_3$ films have been grown under substantial compressive strain, likely suppressing lone pair formation completely. Tensile strains should enhance individual lone pair distortions, but their coupling and resulting long-range order may or may not mimic those found here for bulk samples. Substrate strain values are calculated at room temperature with respect to the experimental cubic lattice parameter of $CsSnBr_3$.

14 Temperature evolution of lattice parameters

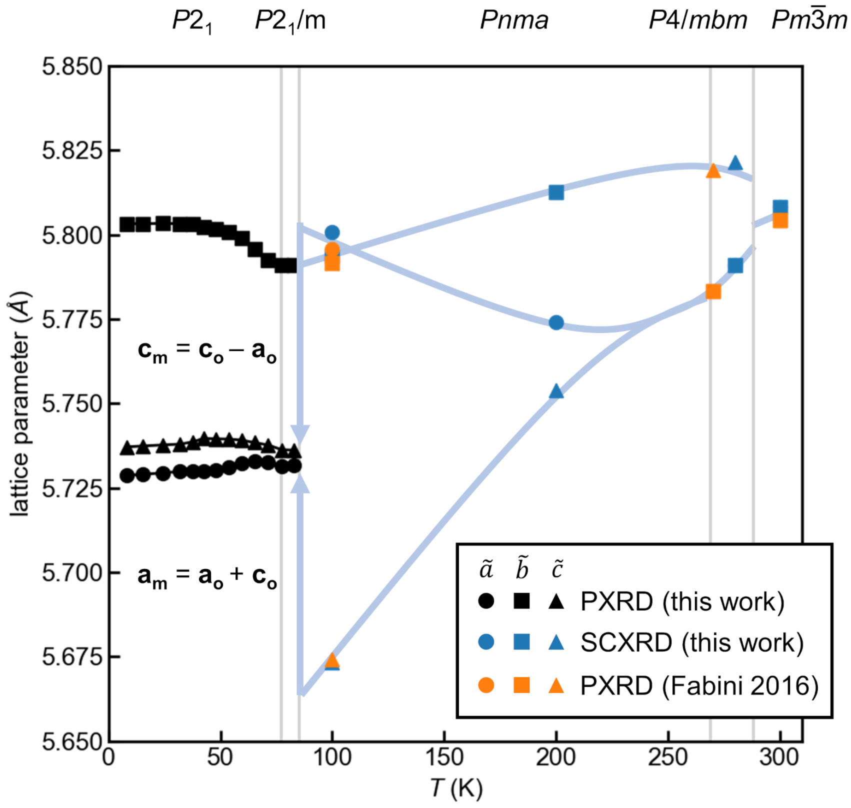

Figure S25: Reduced lattice parameters of $CsSnBr_3$ from sequential Pawley refinement of synchrotron powder diffraction data, from single crystal X-ray diffraction, and from a previous report.[1] The pronounced volumetric negative thermal expansion (NTE) in phase I is dominated by expansion along b, the direction along which the spontaneous polarization develops, suggesting an electrostrictive mechanism as observed in some ferroelectrics. The temperatures of the phase transitions are indicated by gray lines. The apparent jump of a and c lattice parameters at the II–III transition (light blue arrows) is due to the superstructure formation, with the monoclinic a_m and c_m formed from linear combinations of the orthorhombic a_o and c_o which are equivalent in length for $\beta = 90$ deg. Light blue lines are to guide the eye only. Error bars are smaller than the datapoints.

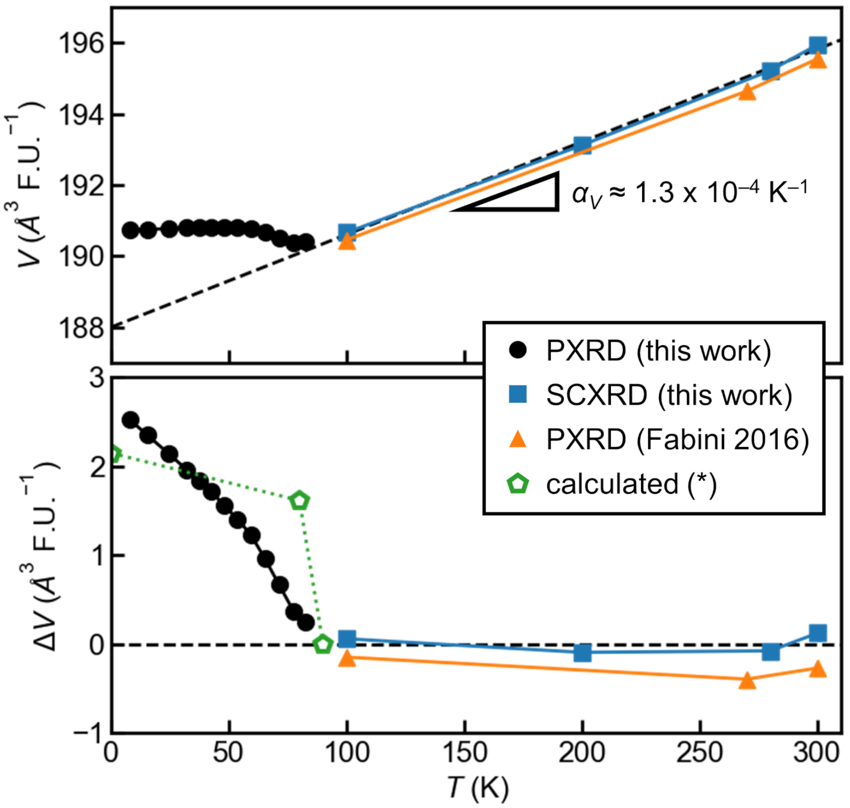

Figure S26: Reduced unit cell volume of $CsSnBr_3$ from sequential Pawley refinement of synchrotron powder diffraction data, from single crystal X-ray diffraction, from a previous report,[1] and from theory. (Top) Reduced volume. A linear fit to the SCXRD data yields the black dashed line. (Bottom) Differential reduced volume. For experimental data, the linear fit from the top panel is subtracted (no attempt is made to handle quantum mechanical deviation from this trend at low temperatures, for lack of data). (*) Calculated data are DFT-optimized, athermal unit cell volumes, plotted at 0 K (phase I), 80 K (phase II), and 90 K (phase III) with respect to the ground state volume of phase III. Reasonable agreement with the observed volume evolution in phases I and II further supports the proposed structure models. Experimental error bars are smaller than the datapoints.

15 Electronic structure impacts of distortions

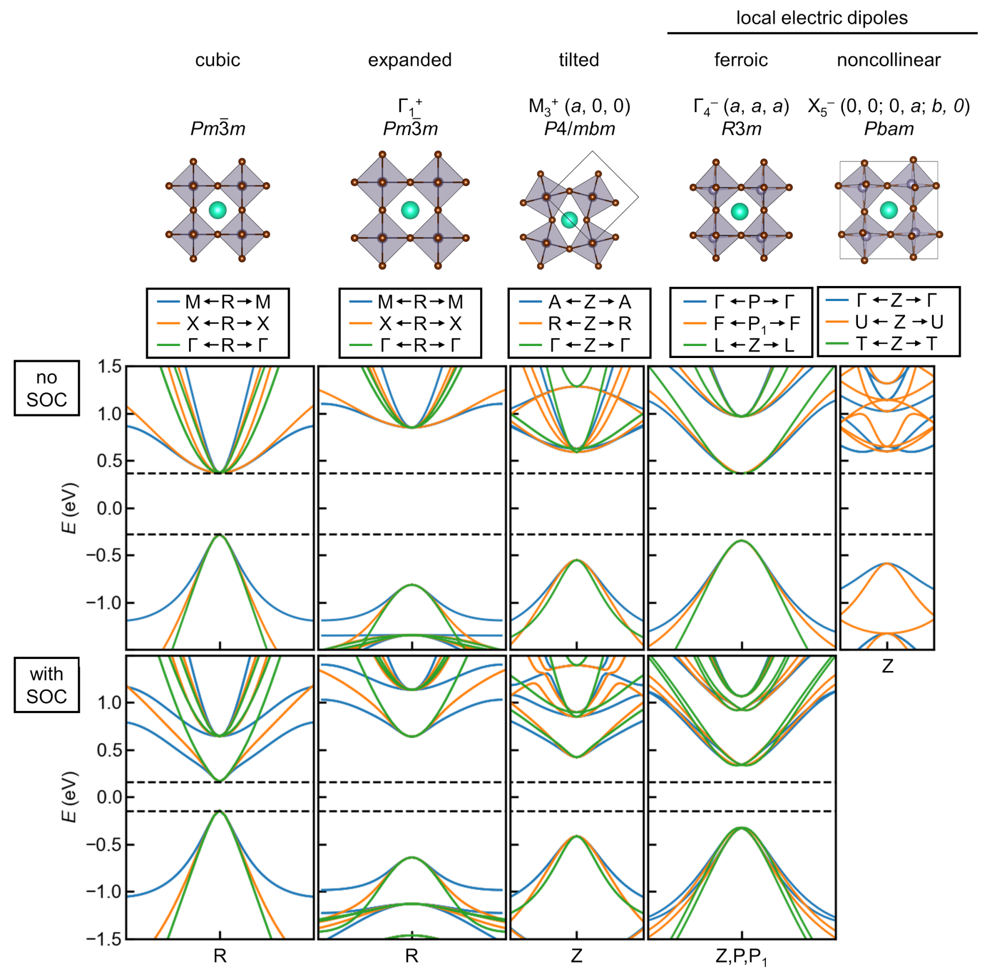

Figure S27: Electronic structure calculations (GGA−PBE) showing the qualitative effects of structural perturbations on the band edge states in $CsSnBr_3$, with and without spin-orbit coupling (SOC). The magnitudes of the distortions are arbitrary. Expansion, in-phase tilting, and zone-center polar distortions ("ferroic," as in $CsGeX_3$) all have the effect of widening the bandgap and enhancing electron and hole effective masses but maintaining a direct bandgap, while zone-boundary noncollinear polar distortions can move the conduction band minimum away from the Z point and produce an indirect gap. Only the ferroic, polar distortion, when SOC is included, splits the valence and conduction band edges in momentum, reflecting bulk Rashba effects in both valence and conduction bands with differing momentum splittings and Rashba coefficients. Given the sharpness of the Fermi-Dirac function at cryogenic temperatures, momentum overlap of excited hole and electron populations leading to the experimentally observed strong photoluminescence attributed to free excitons[29] would seem to be incompatible with a large perturbation of the band edges of either the noncollinear or ferroic (Rashba) type, which is consistent with the small magnitude of the symmetry-breaking distortions from DFT and PXRD. Alternative scenarios are also possible,[30] and the precise electronic structure of phase I requires further study.

16 ^{119}Sn nuclear magnetic resonance (NMR) spectroscopy

Solid-state ^{119}Sn NMR experiments were performed on $CsSnBr_3$ samples prepared from the melt (ground ingot) or from ethylene glycol solution (ground slow-cooled crystals), both neat and after aging in an argon glovebox for 500 days (Figure S26). Interestingly, distinct apparent chemical shifts and lineshapes are observed for the neat samples prepared by the different routes, while powder XRD from the two is indistinguishable (Figure S1). The sample from the melt exhibits a broad, symmetric lineshape ($\delta_{iso} = -289$ ppm), while the crushed, slow-cooled crystals from solution exhibit a sharper, asymmetric lineshape with significant tailing to higher frequency ($\delta_{iso} = -387$ ppm). Kubicki and coworkers reported the ^{119}Sn spectrum of mechanochemically-prepared $CsSnBr_3$ at similar magic angle spinning (MAS) speed.[31] Its apparent chemical shift ($\delta_{iso} = -353$ ppm) is intermediate to those we measure, and it also exhibits tailing to high frequency. Kubicki and coworkers also report the spectrum of β-Sn (a broad signal spanning roughly 6500 ppm to 7500 ppm), consistent with the Knight shift reported by Bloembergen and Rowland (0.74% to 0.79%).[32] Ha and coworkers reported ^{119}Sn spectra of $CH_3NH_3SnI_3$ as a function of air-exposure time.[33] Significant broadening to high frequency was observed over the timescale of hours, and after 6 months air exposure, the signal spanned roughly from 4000 ppm to 0 ppm.

Taken together, these results strongly suggest the apparent chemical shift in these Sn(II) halide perovskites is the sum of a true chemical shift (from chemical shielding in the fictitious absence of free carriers) and a carrier concentration-dependent Knight shift contribution. Under this hypothesis, our melt sample exhibits a symmetric lineshape because it crystallized relatively rapidly in a closed environment (flame-sealed fused silica ampoule) and so exhibits a spatially-uniform free carrier concentration. On the other hand, the slow-cooled crystals from solution were grown over many days under flowing argon, so we hypothesize that the free carrier concentration increases from the center towards the surfaces of the millimeter-scale crystals as the solution ages due to imperfect seals, slight impurity of the inert gas feed, or reactivity with the solvent, leading to the asymmetric lineshape with tailing towards high frequency (the direction of the Knight shift for Sn). The Knight shift is challenging to quantitatively predict,[34] but we note that the $\sim$100 ppm spread in apparent shift between samples prepared by melt, mechanochemical, and solution routes corresponds to about 1.3% of the Knight shift for metallic β-Sn, and reported carrier densities for $CsSnBr_3$ samples prepared by similar methods are several orders of magnitude lower than those for metals like β-Sn.[35] This hypothesis, that perovskite Sn(II) bromides and iodides are such heavily-doped semiconductors that their apparent chemical shifts include a significant Knight shift which is nonetheless small in the usual context of metallic phases, fits with the high and preparation-dependent conductivities observed in these compounds going back to the 1970s, as detailed in the main text.

When the same samples are re-measured after 500 days storage in an argon glovebox, the signals are changed somewhat as shown in Figure S26. Both now exhibit a shifted main peak as well as significant intensity spread over a very broad chemical shift range (as our focus was on the main peak, broadband excitation techniques were not employed and the precise shape and intensity of this broad feature is not quantitative). Experiments with a single spin-echo were also employed to isolate the main peak from this broad feature. Notably, the solution sample, which initially exhibited an asymmetric peak tailed to high frequency, exhibits a more symmetric peak at higher frequency, which could be consistent with equilibration of the free carrier concentration (initially spatially inhomogeneous) throughout the sample. The main peak in the melt sample appears to be slightly shifted to lower frequency upon aging. The nature of the extremely broad background is unclear without further study, though we note its frequency extent is similar to those reported for neat Sn(II) iodide perovskites.[31] The main peaks of the two samples are now more similar in apparent chemical shift, perhaps approaching the same, thermodynamic concentration of point defects in the bulk, with excess impurity atoms possibly annealing out to grain boundaries or extended defects.

While we are confident in the assignment of a preparation-dependent Knight shift contribution to the spectra of $CsSnBr_3$, we are not able to propose a precise interpretation of the inert-aged spectra without further study. Nevertheless, in light of the Knight shift, the changes in the spectra after long-term storage in an inert environment suggest appreciable mobility of the point defects responsible for free carriers (possibly tin vacancies) at room temperature.

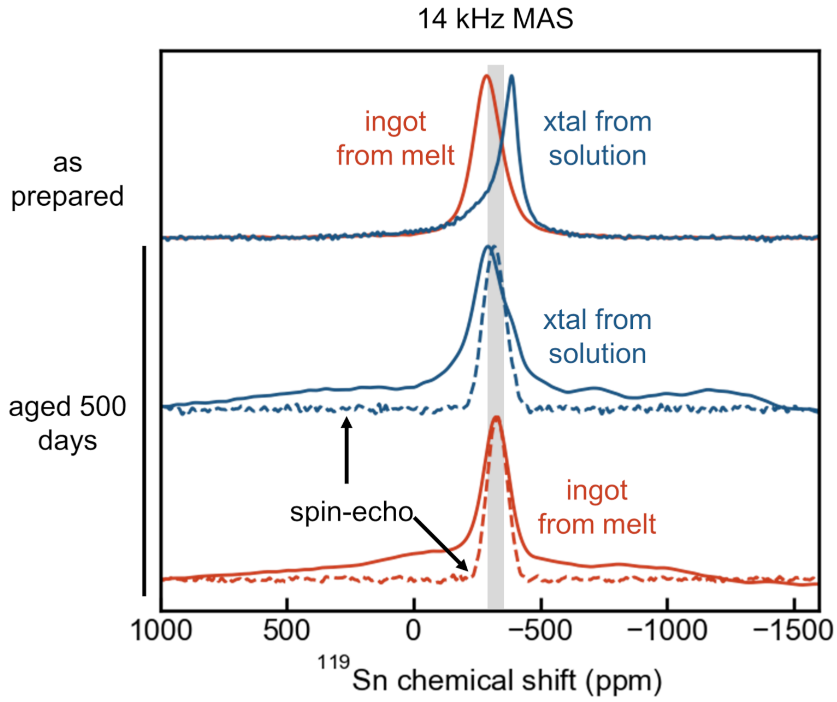

Figure S28: Solid state ^{119}Sn NMR spectra of $CsSnBr_3$. No Sn(IV) impurity phases are detected. Strikingly, samples prepared from the melt or from organic solvents exhibit different chemical shifts despite identical powder diffraction. T_1 times are of the order of milliseconds, and we hypothesize that preparation-dependent free carrier concentrations (from unintentional hole doping) add dissimilar Knight shift terms and hasten spin-lattice relaxation. The unusual asymmetric peak shape for the neat sample from solvent could reflect a changing distribution of point defect concentrations throughout the thickness of the crystal as the solution ages during growth. After 500 days storage in an argon glovebox, the spectra are more complicated, and spin-echo experiments reveal that the components in each sample with the longest spin-spin relaxation time become more similar over time. This suggests mobile point defects which gradually equilibrate to some thermodynamic concentration. The preparation-dependence of the apparent shift is further corroborated by the distinct shift value reported by Kubicki and coworkers.[31]

17 Electronic conductivity comparison between $CsGeBr_3$, $CsSnBr_3$, and $CsPbBr_3$

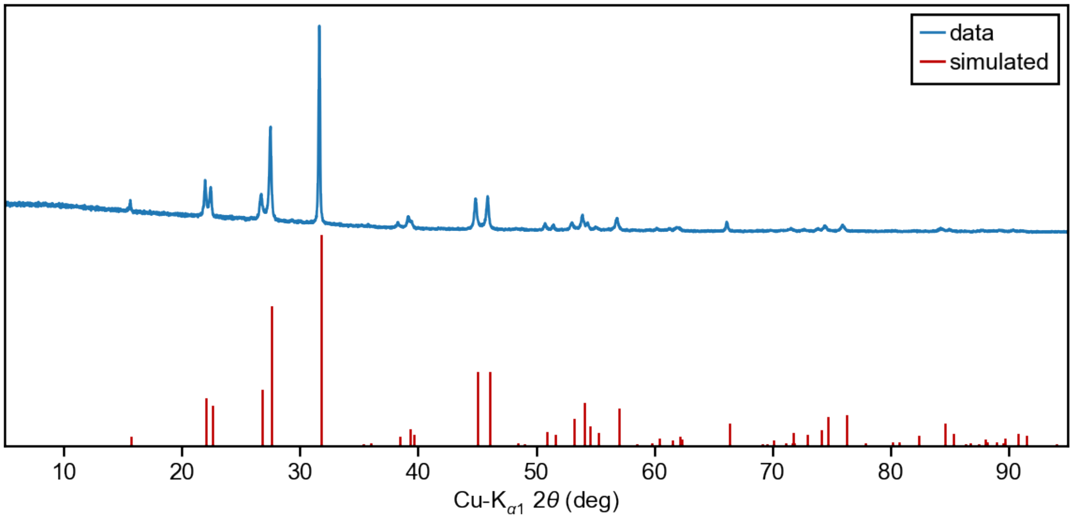

Figure S29: Powder X-ray diffraction (Cu-K$_{\alpha1}$) for crushed $CsGeBr_3$ crystals grown from hydrobromic/hypophosphorous acid solution. Red ticks indicate the Bragg reflection angles and intensities expected for the reported room temperature, rhombohedral phase.[14]

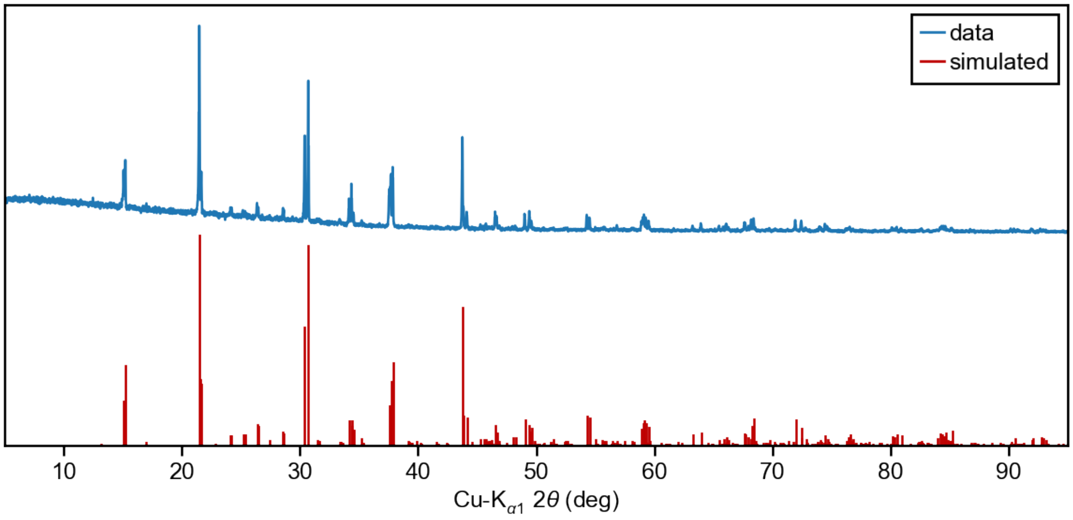

Figure S30: Powder X-ray diffraction (Cu-K$_{\alpha1}$) for $CsPbBr_3$ precipitated from hydrobromic acid solution. Red ticks indicate the Bragg reflection angles and intensities expected for the reported room temperature, orthorhombic phase.[21]

The equivalent AC conductivity, σ_{AC}, for comparison of $CsGeBr_3$, $CsSnBr_3$, and $CsPbBr_3$ is given as

$$\sigma_{AC} = \frac{L}{A}\mathrm{Re}\left(\frac{1}{Z}\right), \quad (6)$$

where L is the pellet thickness, A is the pellet cross-sectional area, and Z is the measured (complex) impedance. This formulation ignores the contact resistance, which is of order 10^1 Ω cm, which does not affect the qualitative conclusion here that the resistivities of the three compounds are separated by many orders of magnitude, and are non-monotonic with variation of the group 14 cation, with $CsSnBr_3$ the most conductive by far.

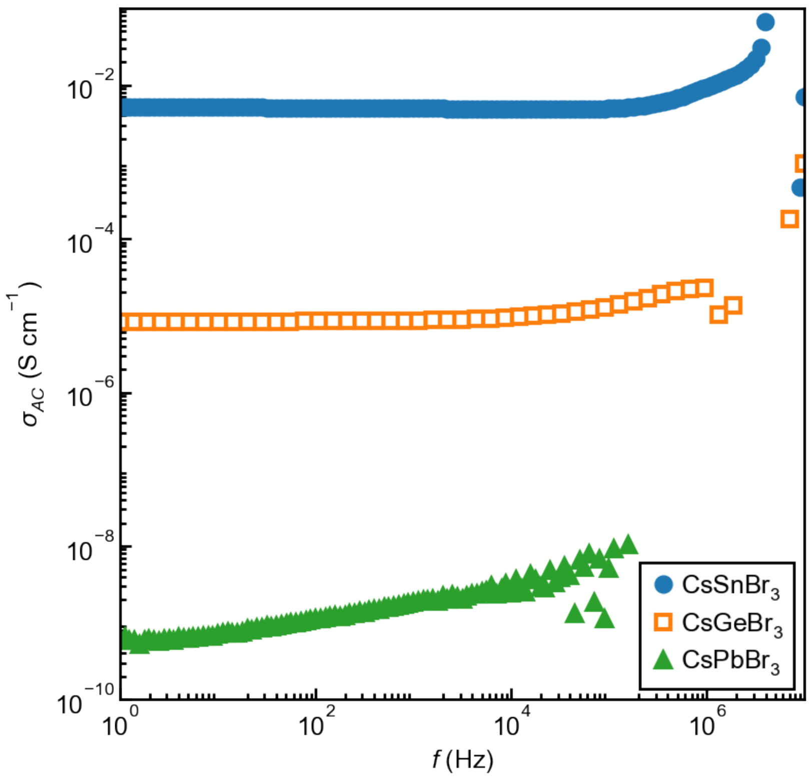

Figure S31: Equivalent AC conductivity, σ_{AC}, extracted from impedance spectroscopy.

References

[1] Fabini, D. H.; Laurita, G.; Bechtel, J. S.; Stoumpos, C. C.; Evans, H. A.; Kontos, A. G.; Raptis, Y. S.; Falaras, P.; der Ven, A. V.; Kanatzidis, M. G.; Seshadri, R. Dynamic Stereochemical Activity of the Sn^{2+} Lone Pair in Perovskite $CsSnBr_3$. *J. Am. Chem. Soc.* **2016**, *138*, 11820–11832.

[2] Pallikara, I.; Kayastha, P.; Skelton, J. M.; Whalley, L. D. The physical significance of imaginary phonon modes in crystals. *Electron. Struct.* **2022**, *4*, 033002.

[3] de Faria, J. L.; Hellner, E.; Liebau, F.; Makovicky, E.; Parthé, E. Nomenclature of inorganic structure types. Report of the International Union of Crystallography Commission on Crystallographic Nomenclature Subcommittee on the Nomenclature of Inorganic Structure Types. *Acta Crystallogr. A* **1990**, *46*, 1–11.

[4] Stokes, H. T.; Kisi, E. H.; Hatch, D. M.; Howard, C. J. Group-theoretical analysis of octahedral tilting in ferroelectric perovskites. *Acta Crystallogr. B: Struct. Sci. Cryst. Eng. Mater.* **2002**, *58*, 934–938.

[5] Bärnighausen, H. Group-subgroup relations between space groups: a useful tool in crystal chemistry. *Commun. Math. Chem.* **1980**, *9*, 139–175.

[6] Müller, U. Kristallographische Gruppe-Untergruppe-Beziehungen und ihre Anwendung in der Kristallchemie. *Z. Anorg. Allg. Chem.* **2004**, *630*, 1519–1537.

[7] Müller, U.; Wondratschek, H. International Tables for Crystallography, Vol. A1, Symmetry relations between space groups. John Wiley & Sons, Chichester, United Kingdom, 2010.

[8] Müller, U. *Symmetriebeziehungen zwischen verwandten Kristallstrukturen, 2nd Edition*; Springer Spektrum: Berlin, Heidelberg, Germany, 2023.

[9] Bock, O.; Müller, U. Symmetrieverwandtschaften bei Varianten des Perowskit-Typs. *Acta Crystallogr. B* **2002**, *58*, 594–606.

[10] Tran, T. T.; Halasyamani, P. S. Synthesis and characterization of $ASnF_3$ ($A=Na^+$, K^+, Rb^+, Cs^+). *J. Solid State Chem.* **2014**, *210*, 213–218.

[11] Poulsen, F. R.; Rasmussen, S. E.; Haaland, A.; Jerslev, B.; Schäffer, C. E.; Sunde, E.; Sørensen, N. A. Crystal Structure and Phase Transition of Cesium Trichlorostannate(II). *Acta Chem. Scand.* **1970**, *24*, 150–156.

[12] Yamada, K.; Fujise, K.; Hino, S.; Yamane, Y.; Nakagama, T. Characterization of Sn(II)-based Perovskites by XRD, DTA, NQR and ^{119}Sn NMR for Photovoltaic Applications. *Chem. Lett.* **2019**, *48*, 749–752.

[13] Swainson, I.; Chi, L.; Her, J.-H.; Cranswick, L.; Stephens, P.; Winkler, B.; Wilson, D. J.; Milman, V. Orientational ordering, tilting and lone-pair activity in the perovskite methylammonium tin bromide, $CH_3NH_3SnBr_3$. *Acta Crystallogr. B: Struct. Sci. Cryst. Eng. Mater.* **2010**, *66*, 422–429.

[14] Thiele, G.; Rotter, H. W.; Schmidt, K. D. Kristallstrukturen und Phasentransformationen von Caesiumtrihalogenogermanaten(II) $CsGeX_3$ (X = Cl, Br, I). *Z. Anorg. Allg. Chem.* **1987**, *545*, 148–156.

[15] Mauersberger, P.; Huber, F. Structure of caesium triiodostannate(II). *Acta Crystallogr. B* **1980**, *36*, 683–684.

[16] Yamada, K.; Funabiki, S.; Horimoto, H.; Matsui, T.; Okuda, T.; Ichiba, S. Structural Phase Transitions of the Polymorphs of $CsSnI_3$ by Means of Rietveld Analysis of the X-Ray Diffraction. *Chem. Lett.* **1991**, *20*, 801–804.

[17] Berastegui, P.; Hull, S.; Eriksson, S.-G. A low-temperature structural phase transition in $CsPbF_3$. *J. Phys. Condens. Matter* **2001**, *13*, 5077–5088.

[18] Cape, J. A.; White, R. L.; Feigelson, R. S. EPR Study of the Structure of $CsPbCl_3$. *J. Appl. Phys.* **1969**, *40*, 5001–5005.

[19] Cohen, M. I.; Young, K. F.; Chang, T.-T.; Brower, W. S. Phase Transitions in $CsPbCl_3$. *J. Appl. Phys.* **1971**, *42*, 5267–5272.

[20] Hidaka, M.; Okamoto, Y.; Zikumaru, Y. Structural Phase Transition of $CsPbCl_3$ below Room Temperature. *Phys. Status Solidi* **1983**, *79*, 263–269.

[21] Rodová, M.; Brožek, J.; Knížek, K.; Nitsch, K. Phase transitions in ternary caesium lead bromide. *J. Therm. Anal. Calorim.* **2003**, *71*, 667–673.

[22] Trots, D.; Myagkota, S. High-temperature structural evolution of caesium and rubidium triiodoplumbates. *J. Phys. Chem. Solids* **2008**, *69*, 2520–2526.

[23] Sutton, R. J.; Filip, M. R.; Haghighirad, A. A.; Sakai, N.; Wenger, B.; Giustino, F.; Snaith, H. J. Cubic or Orthorhombic? Revealing the Crystal Structure of Metastable Black-Phase $CsPbI_3$ by Theory and Experiment. *ACS Energy Lett.* **2018**, *3*, 1787–1794.

[24] Yamada, K.; Nose, S.; Umehara, T.; Okuda, T.; Ichiba, S. ^{81}Br NQR and ^{119}Sn Mössbauer Study for $MSnBr_3$ (M=Cs and CH_3NH_3). *Bull. Chem. Soc. Jpn.* **1988**, *61*, 4265–4268.

[25] Pyykkö, P. Year-2017 nuclear quadrupole moments. *Mol. Phys.* **2018**, *116*, 1328–1338.

[26] Swift, M. W.; Lyons, J. L. Lone-Pair Stereochemistry Induces Ferroelectric Distortion and the Rashba Effect in Inorganic Halide Perovskites. *Chem. Mater.* **2023**, *35*, 9370–9377.

[27] Porezag, D.; Pederson, M. R. Infrared intensities and Raman-scattering activities within density-functional theory. *Phys. Rev. B* **1996**, *54*, 7830–7836.

[28] Goldstein, D. H. *Polarized Light*; CRC Press, 2017.

[29] Tan, J.; Zhou, Y.; Lu, D.; Feng, X.; Liu, Y.; Zhang, M.; Lu, F.; Huang, Y.; Xu, X. Temperature-dependent photoluminescence of lead-free cesium tin halide perovskite microplates. *Chin. Phys. B* **2023**, *32*, 117802.

[30] Becker, M. A. et al. Bright triplet excitons in caesium lead halide perovskites. *Nature* **2018**, *553*, 189–193.

[31] Kubicki, D. J.; Prochowicz, D.; Salager, E.; Rakhmatullin, A.; Grey, C. P.; Emsley, L.; Stranks, S. D. Local Structure and Dynamics in Methylammonium, Formamidinium, and Cesium Tin(II) Mixed-Halide Perovskites from ^{119}Sn Solid-State NMR. *J. Am. Chem. Soc.* **2020**, *142*, 7813–7826.

[32] Bloembergen, N.; Rowland, T. On the nuclear magnetic resonance in metals and alloys. *Acta Metall.* **1953**, *1*, 731–746.

[33] Ha, M.; Karmakar, A.; Bernard, G. M.; Basilio, E.; Krishnamurthy, A.; Askar, A. M.; Shankar, K.; Kroeker, S.; Michaelis, V. K. Phase Evolution in Methylammonium Tin Halide Perovskites with Variable Temperature Solid-State ^{119}Sn NMR Spectroscopy. *J. Phys. Chem. C* **2020**, *124*, 15015–15027.

[34] d'Avezac, M.; Marzari, N.; Mauri, F. Spin and orbital magnetic response in metals: Susceptibility and NMR shifts. *Phys. Rev. B* **2007**, *76*.

[35] Zhang, Y.; Yao, Q.; Qian, J.; Zhao, X.; Li, D.; Mi, Q. Thermoelectric properties of all-inorganic perovskite $CsSnBr_3$: A combined experimental and theoretical study. *Chem. Phys. Lett.* **2020**, *754*, 137637.
